# Metabolic dysfunction in women with PCOS is more closely linked to BMI than to hyperandrogenism

**DOI:** 10.3389/fendo.2026.1923923

**Published:** 2026-08-31

**Authors:** Paria Naderi, Birte Hellwig, Lukas van Baal, Alice Schmidt, Julia Messner, Dagmar Führer, Theresia Sarabhai

**Affiliations:** 1 Department of Endocrinology, Diabetology and Metabolism, Medical Faculty and University Hospital Essen, University of Duisburg-Essen, Essen, Germany; 2 Institute for Medical Informatics, Biometry and Epidemiology, University Hospital Essen, University of Duisburg-Essen, Essen, Germany

**Keywords:** glucose exposure, HOMA-IR, hyperandrogenism, insulin resistance, matsuda index, metabolic phenotyping, obesity, PCOS

## Abstract

**Introduction:**

Polycystic ovary syndrome (PCOS) is frequently conceptualized as a hyperandrogenic disorder, yet cardiometabolic risk may be driven primarily by obesity and insulin resistance. We characterized glucose–insulin dynamics in women with PCOS and examined whether metabolic dysfunction was more closely associated with obesity status than with androgen-related parameters.

**Methods:**

In this retrospective cross-sectional study, 71 women with PCOS underwent a standardized 3-hour oral glucose tolerance test (OGTT, 0–180 min) and were stratified by obesity status (BMI <30 kg/m², n=37; BMI ≥30 kg/m², n=34). OGTT-derived indices included the Matsuda index, HOMA-IR and AUCglucose_(0–180)_. Associations were assessed using Pearson correlations and parsimonious multivariable linear regression models for log(Matsuda), log(HOMA-IR), and AUCglucose_(0–180)_ including BMI, age and total testosterone as predictors.

**Results:**

Obese women were older and had higher fasting glucose than non-obese women, whereas HbA1c was only numerically higher. During the OGTT, they showed higher glucose and insulin concentrations, lower insulin sensitivity, higher fasting insulin resistance, greater glucose exposure, and a higher insulin response relative to glucose exposure. PCOM prevalence, Rotterdam phenotype distribution, moderate-to-severe hirsutism and menstrual grades were similar between groups. Total testosterone and adrenal androgens were comparable. Across the parsimonious model, BMI was the only consistent independent predictor of log(Matsuda), log(HOMA-IR), and AUCglucose_(0–180)_.

**Conclusions:**

Within this PCOS cohort, metabolic impairment was more closely associated with BMI and obesity status than with the androgen-related parameters assessed. These findings support metabolic risk stratification in PCOS focused on obesity and insulin resistance rather than broader androgen-related markers.

## Introduction

Polycystic ovary syndrome (PCOS) is among the most common endocrine disorders in women of reproductive age and is diagnosed in adults by the presence of two of three core features: clinical and/or biochemical hyperandrogenism, ovulatory dysfunction, and polycystic ovarian morphology (PCOM) (1). Although PCOS is often framed as a reproductive or hyperandrogenic disorder, current guidelines emphasize its broader multisystem nature, including metabolic manifestations and increased cardiometabolic risk across the lifespan (1, 2).

The cardiometabolic burden associated with PCOS is substantial. Longitudinal evidence indicates increased risks of hypertension, type 2 diabetes, adverse lipid profiles, and non-fatal cerebrovascular events in women with PCOS compared with women without PCOS (3). In a meta-analysis of longitudinal studies, PCOS was associated with risk ratios of 1.75 for hypertension, 3.00 for type 2 diabetes, and 1.41 for non-fatal cerebrovascular events (3). Earlier meta-analytic data also showed increased odds of impaired glucose tolerance, type 2 diabetes, and metabolic syndrome of 2.48, 4.43, and 2.88, respectively (4). These findings underscore the importance of identifying which features of PCOS are most closely linked to metabolic risk.

Insulin resistance is a metabolic feature that may contribute to the association between PCOS and adverse metabolic outcomes (5). Prospective data suggest that progression from PCOS to dysglycaemia is clinically meaningful over time (5). In one long-term prospective study, the cumulative incidence of type 2 diabetes reached 16.9%, and baseline BMI, fasting glucose, and glucose area under the curve emerged as independent predictors of diabetes development (6). These observations are particularly relevant for studies using oral glucose tolerance testing, as dynamic glucose-insulin phenotyping may detect early abnormalities not captured by fasting measures alone.

Obesity is highly prevalent in PCOS and clearly amplifies its metabolic burden, yet it does not fully explain the phenotype. Approximately half of women with PCOS are obese in clinical cohorts, and a meta-analysis reported increased risks of overweight, obesity, and central obesity, with relative risks of 1.95, 2.77, and 1.73, respectively (3, 7). At the same time, insulin resistance and adipose tissue dysfunction may also be present in the absence of overt excess adiposity (8, 9). Mechanistically, adipose tissue dysfunction in PCOS has been linked to impaired insulin-mediated glucose transport, dysregulated lipolysis, altered adipokine secretion, inflammation, and ectopic fat-related metabolic stress (9).

Nevertheless, whether hyperandrogenism independently drives metabolic dysfunction in PCOS remains unresolved. Hyperandrogenism is central to the clinical identity of PCOS, and bidirectional interactions between insulin excess and androgen production are biologically plausible (2). Some studies suggest that hyperandrogenic and/or anovulatory phenotypes exhibit more severe metabolic impairment, whereas others indicate that body weight may outweigh Rotterdam phenotype in determining metabolic status (2, 10). Because androgen excess and adiposity frequently coexist in PCOS, it remains difficult to disentangle whether metabolic dysfunction is primarily linked to hyperandrogenism itself or instead reflects obesity-associated insulin resistance (8)Supporting the latter interpretation, recent work suggests that dynamic metabolic abnormalities may align more closely with insulin resistance than with hyperandrogenism per se (11).

In this retrospective cross-sectional analysis, we characterized glucose-insulin dynamics in a cohort of women with PCOS using a standardized 3-hour oral glucose tolerance test (OGTT) and examined whether metabolic dysfunction was more closely associated with obesity status and BMI than with androgen-related parameters. By comparing non-obese and obese women and integrating dynamic OGTT-derived indices with multivariable models, we aimed to clarify whether the adverse metabolic phenotype in PCOS is primarily linked to hyperandrogenism or instead reflects adiposity-associated insulin resistance.

## Methods

### Study design and study population

This retrospective cross-sectional study included adult women with PCOS who were evaluated at the outpatient clinic of the Department of Endocrinology, Diabetology and Metabolism, University Hospital Essen, Germany, between January 2016 and December 2024. PCOS was diagnosed according to the Rotterdam criteria, requiring the presence of at least two of the following three features after exclusion of related differential diagnoses: clinical and/or biochemical hyperandrogenism, oligo-/anovulation, and PCOM confirmed based on mandatory ultrasound reports. Based on these features, participants were classified into Rotterdam phenotype A (hyperandrogenism + oligo-/anovulation + PCOM), phenotype B (hyperandrogenism + oligo-/anovulation), phenotype C (hyperandrogenism + PCOM), or phenotype D (oligo-/anovulation + PCOM) (**Supplementary Table 1**) (1, 2). For the present analysis, women were eligible if they were aged ≥18 years, had undergone a standardized 75-g 3-hour OGTT, and had a comprehensive endocrine and metabolic assessment including androgen-related laboratory parameters. Known diabetes mellitus was an exclusion criterion. To avoid confounding of reproductive hormone and androgen measurements, women using oral contraceptives were excluded from the analysis. The final study population comprised 71 women and was stratified according to obesity status into a non-obese group (body mass index [BMI] <30.0 kg/m², n=37) and an obese group (BMI ≥30.0 kg/m², n=34) (**Supplementary Figure 1**).

### Clinical and anthropometric assessment

Clinical and anthropometric data were obtained from the medical records at the time of metabolic phenotyping. Clinical hyperandrogenism was assessed using the Ferriman–Gallwey score and categorized into severity groups according to the clinical dataset (12). Female pattern hair loss was documented according to the Ludwig classification (13). Menstrual cycle disturbance was graded clinically on an ordinal scale as follows: grade 0, normal cycle length (28–35 days); grade 1, mildly prolonged cycle length (>35 to 60 days); grade 2, markedly prolonged cycle length (>60 days); and grade 3, secondary amenorrhea (chronic amenorrhea). PCOM was documented according to the ultrasound reports. Medication use at the time of assessment, including metformin, levothyroxine, and beta-blockers, was documented from the clinical records. Cycle day at OGTT was recorded from the clinical documentation and evaluated in change-in-estimate analyses. Because the physiological interpretation of a recorded cycle day is limited in women with oligo- or amenorrhea, cycle day was not included in the primary multivariable models. Reproductive and androgen-related parameters were obtained from routine clinical assessment; free and bioavailable testosterone were calculated rather than directly measured, and PCOM classification was based on ultrasound reports.

### Laboratory assessment

Venous blood samples were obtained after a 12-hour overnight fast as part of routine clinical work-up. Laboratory parameters included fasting glucose, glycated hemoglobin (HbA1c), insulin, C-peptide, thyroid-stimulating hormone (TSH), free thyroxine (fT4), luteinizing hormone (LH), follicle-stimulating hormone (FSH), prolactin, progesterone, estradiol, total testosterone, sex hormone-binding globulin (SHBG), dehydroepiandrosterone sulfate (DHEA-S), androstenedione, 17-hydroxyprogesterone, and anti-Müllerian hormone (AMH). Free and bioavailable testosterone were calculated according to the Vermeulen formula (14). Except for samples analyzed externally, blood samples were immediately transferred to the laboratory department of University Hospital Essen. Detailed information on the biochemical analyses is provided in the **Supplementary Material**.

### Oral glucose tolerance test and derived indices

All participants underwent a standardized 75-g OGTT after a 12-hour overnight fast. Venous blood samples were collected at 0, 30, 60, 90, 120 and 180 minutes for measurement of plasma glucose, serum insulin, and C-peptide. Whole-body insulin sensitivity was estimated using the Matsuda index, and fasting insulin resistance was assessed by the homeostatic model assessment for insulin resistance (HOMA-IR). Glycemic responses during the OGTT were quantified by calculating the area under the curve from 0 to 180 minutes for glucose and insulin, as well as the AUCins/AUCglu ratio. Detailed information on OGTT-derived indices is provided in the **Supplementary Material**.

### Statistical analysis

Participants were analyzed according to obesity status (BMI <30.0 vs ≥30.0 kg/m²). Continuous variables are presented as mean ± standard deviation (SD) or median (interquartile range, IQR), as appropriate, and categorical variables as absolute counts and percentages. Because of missing values, the number of observations could vary across variables and is indicated in the tables where applicable. Missing data were limited to AMH in four participants, SHBG in one participant, which also prevented calculation of free and bioavailable testosterone, DHEA-S in one participant, and C-peptide in one participant. Analyses were performed using available observations, and the corresponding sample sizes are reported in the tables. Between-group comparisons were performed using unpaired Student’s t-test or Mann–Whitney U test for continuous variables and chi-square test or Fisher’s exact test for categorical variables, as appropriate. Exploratory comparisons of metabolic outcomes according to Ferriman–Gallwey score category, Ludwig-classified alopecia, and hyperandrogenic Rotterdam phenotype were performed using two-sided Mann–Whitney U tests (**Supplementary Table 2**).

Exploratory pooled correlation analyses and univariable linear regression models were performed to assess associations between candidate predictors and metabolic outcomes. Univariable models were fitted for log-transformed Matsuda index, log-transformed HOMA-IR, and AUCglucose_(0–180)_ using all available observations for each predictor-outcome combination. Multivariable linear regression models were used to assess independent determinants of metabolic dysfunction. Because of their right-skewed distributions and to improve model assumptions, the Matsuda index and HOMA-IR were natural log-transformed before modeling.

Based on the *a priori* study hypothesis addressing the relative contributions of adiposity and androgen-related parameters, the primary parsimonious models included BMI, age, and total testosterone as simultaneous predictors of log-transformed Matsuda index, log-transformed HOMA-IR, and AUCglucose_(0–180)_. To permit direct comparison with the corresponding broader models, the primary parsimonious models were fitted in the same complete-case sample of 67 participants.

Broader multivariable models included BMI, age, TSH, androstenedione, AMH, and either total or calculated free testosterone and were retained as sensitivity analyses to assess whether reducing the number of predictors materially affected the parameter estimates (**Supplementary Tables 3**, **S4**).

In sensitivity analyses, total testosterone was replaced by calculated free testosterone in otherwise identical parsimonious models. These analyses were performed in 66 participants because SHBG, which is required for calculation of free testosterone, was unavailable in one participant (**Supplementary Table 5**).

Model assumptions were assessed using residuals-versus-fitted, normal Q–Q, scale-location, and residuals-versus-leverage plots, including Cook’s distance (**Supplementary Figures 3–S6**).

Model fit was summarized using the adjusted coefficient of determination (adjusted R²) (**Supplementary Table 6**). An additional sensitivity analysis of the broader AUCglucose model was performed after excluding two observations identified as potentially influential in the diagnostic plots (**Supplementary Table 7**).

Additional supplementary analyses included univariable regression models for candidate predictors (**Supplementary Table 8**). Change-in-estimate analyses were performed by adding potential confounders individually to the parsimonious main models including BMI, age, and directly measured total testosterone. The percentage change in the BMI coefficient relative to the corresponding parsimonious main model was calculated (**Supplementary Table 9**).

Statistical analyses and graphical visualization were performed using R 4.5.0 (R Core Team, R Foundation for Statistical Computing, Vienna, Austria) and GraphPad Prism, version 11.0.0 (93) (GraphPad Software, San Diego, CA, USA). All tests were two-sided, and a p-value <0.05 was considered statistically significant. More detailed information on statistical analyses is provided in the **Supplementary Material**.

## Results

### Baseline clinical, endocrine, and metabolic characteristics according to obesity status

A total of 71 women with PCOS were included in the obesity-stratified analysis and categorized into a non-obese group (BMI <30 kg/m², n = 37) and an obese group (BMI ≥30 kg/m², n = 34) (**Table 1**). As expected, obese participants had a markedly higher BMI than non-obese participants (37.0 ± 5.1 vs 23.8 ± 2.9 kg/m², p < 0.0005). The obese group was also older (31.8 ± 7.2 vs 28.1 ± 7.3 years, p=0.037). Fasting blood glucose was higher in obese women (96.8 ± 11.0 vs 91.6 ± 7.9 mg/dL, p=0.026), whereas HbA1c was only numerically higher and did not reach statistical significance (5.5 ± 0.3 vs 5.3 ± 0.3%, p=0.074). Thyroid function parameters (TSH, fT4) were comparable between groups. PCOM prevalence and the proportion of hyperandrogenic Rotterdam phenotypes A-C were similar between groups. Moderate-to-severe hirsutism, defined as a Ferriman–Gallwey score ≥17, was numerically more frequent in obese than in non-obese women (35% vs 22%), but the difference was not statistically significant (p = 0.291). The prevalence of Ludwig-classified alopecia was also comparable between groups (9% vs 11%, p > 0.99), and the overall distribution of menstrual disturbance grades did not differ significantly (p = 0.373) (**Table 1**; **Supplementary Table 1**).

**Table 1:** Clinical and anthropometric characteristics, medication use, and fasting laboratory parameters in women with PCOS stratified by obesity status (non-obese, BMI < 30 kg/m²; obese ≥30 kg/m²).

Variable	Non-obese	Obese	P-value
n	37	34	
Age (years)	28.1 ± 7.3	31.8 ± 7.2	0.037
BMI (kg/m²)	23.8 ± 2.9	37.0 ± 5.1	<0.0005
PCOM (yes/no) [%]	29/8 [78/22]	29/5 [85/15]	0.547
Ferriman-Gallwey score (≥17) [%]	8 [22]	12 [35]	0.291
Ludwig-classified alopecia (yes/no) [%]	4/33 [11/89]	3/31 [9/91]	>0.99
Hyperandrogenic PCOS phenotype (A/B/C) [%]	33 [89]	28 [82]	0.504
Metformin use, n [%]	9 [24]	15 [44]	0.087
Levothyroxine use, n [%]	6 [16]	14 [41]	0.033
Beta-blocker use, n [%]	2 [5]	4 [12]	0.417
Menstrual disturbance grade (0/1/2/3) [%]	13/15/5/4 [35/41/14/11]	10/10/5/9 [29/29/14/26]	0.373
Fasting blood glucose (mg/dL)	91.6 ± 7.9	96.8 ± 11.0	0.026
HbA1c (%)	5.3 ± 0.3	5.5 ± 0.3	0.074
TSH (mU/L)	2.0 (1.3–3.0)	1.9 (1.3–2.8)	0.589
fT4 (pmol/L)	14.5 ± 2.0	15.0 ± 1.9	0.219
LH (IU/L)	7.9 (5.0-12.4)	5.5 (3.8-8.1)	0.064
FSH (IU/L)	5.8 (4.2-6.9)	5.2 (3.7-6.3)	0.203
LH/FSH ratio	1.3 (1.0-2.0)	1.3 (0.7-1.6)	0.115
Total testosterone (nmol/L)	1.3 ± 0.3	1.3 ± 0.5	0.815
Bioavailable testosterone (nmol/L)	0.4 ± 0.2	0.5 ± 0.3	0.038
Free testosterone (pmol/L)	16.0 ± 9.2	21.9 ± 13.6	0.038
SHBG (nmol/L)	58.4 (34.6–98.7)	33.3 (21.4-58.0)	0.191
DHEA-S (µg/dL)	207.8 ± 85.1	227.6 ± 160.1	0.517
Androstenedione (ng/mL)	3.1 ± 1.4	3.0 ± 1.6	0.831
17-OH-progesterone (ng/dL)	147 (130.0-198.0)	125 (85.0-208.5)	0.873
Prolactin (ng/mL)	10.1 (7.6-14.5)	10.9 (8.1-17.5)	0.423
Progesterone (ng/mL)	0.8 (0.5–2.0)	0.7 (0.5-1.1)	0.606
Estradiol (pg/mL)	61.3 (45.7–130.1)	53.9 (38.5–85.2)	0.024
AMH (µg/L)	4.2 (3.2–7.3)	3.8 (2.4-5.5)	0.043

Data are presented as mean ± SD, median (IQR) for skewed variables, and n (%) for categorical variables, as appropriate. Percentages are shown by category in brackets. Participants were assessed after a 12-hour overnight fast. BMI categories were defined as non-obese (BMI <30 kg/m²) and obese (BMI ≥30 kg/m²). Continuous variables were compared using unpaired Student’s t-tests or Mann–Whitney U tests, depending on their distribution. Categorical variables were compared using chi-square tests or Fisher’s exact tests, as appropriate; the overall distribution of menstrual-disturbance grades was compared using a global chi-square test. All p-values are two-sided.

Polycystic ovarian morphology (PCOM) was recorded as present/absent based on ultrasound reports (yes/no). Moderate-to-severe hirsutism was defined as a Ferriman–Gallwey score ≥17. Female pattern hair loss was classified as present or absent according to the Ludwig classification. Hyperandrogenic PCOS phenotypes comprised Rotterdam phenotypes A–C, whereas phenotype D was classified as non-hyperandrogenic. Menstrual disturbance was graded as follows: grade 0, eumenorrhea with a cycle length of 28–35 days; grade 1, mildly prolonged cycle length of >35–60 days; grade 2, markedly prolonged cycle length of >60 days; and grade 3, secondary amenorrhea. Free and bioavailable testosterone were calculated according to the Vermeulen formula.

Data were available for 67 participants for AMH and for 70 participants for SHBG, calculated free and bioavailable testosterone, and DHEA-S; the remaining variables shown in the table had complete data.

AMH, anti-Müllerian hormone; BMI, body mass index; DHEA-S, dehydroepiandrosterone sulfate; FSH, follicle-stimulating hormone; fT4, free thyroxine; HbA1c, glycated hemoglobin A1c; IQR, interquartile range; LH, luteinizing hormone; PCOM, polycystic ovarian morphology; PCOS, polycystic ovary syndrome; SD, standard deviation; SHBG, sex hormone-binding globulin; TSH, thyroid-stimulating hormone.

Metformin use was numerically more frequent in obese women (44% vs 24%, p = 0.087). Levothyroxine use was significantly more common in obese than in non-obese women (41% vs 16%, p = 0.033), whereas beta-blocker use did not differ significantly between groups (12% vs 5%, p = 0.417).

Among androgen-related parameters, free testosterone (21.9 ± 13.6 vs 16.0 ± 9.2 pmol/L, p=0.038) and bioavailable testosterone (0.5 ± 0.3 vs 0.4 ± 0.2 nmol/L, p=0.038) were higher in obese women. Total testosterone, DHEA-S, and androstenedione were similar between groups. SHBG was numerically lower in obese women (33.3 [21.4–58.0] vs 58.4 [34.6–98.7] nmol/L, p=0.191). Estradiol (53.9 [38.5–85.2] vs 61.3 [45.7–130.1] pg/mL, p = 0.024) and AMH (3.8 [2.4–5.5] vs 4.2 [3.2–7.3] µg/L, p = 0.043) were lower in obese women (**Table 1**). Descriptive distributions of LH, FSH, progesterone, and estradiol across recorded cycle-day categories are shown in **Supplementary Figure 2**, with the most pronounced between-category variation observed for progesterone and estradiol; no formal statistical comparisons were performed. In exploratory analyses, no statistically significant differences in log(Matsuda), log(HOMA-IR), or AUCglucose_(0–180)_ were observed according to Ferriman–Gallwey category, Ludwig-classified alopecia, or hyperandrogenic Rotterdam phenotype (**Supplementary Table 2**).

### Obesity is associated with a higher insulin-to-glucose response, reduced insulin sensitivity, and increased fasting insulin resistance

**Figure 1:**
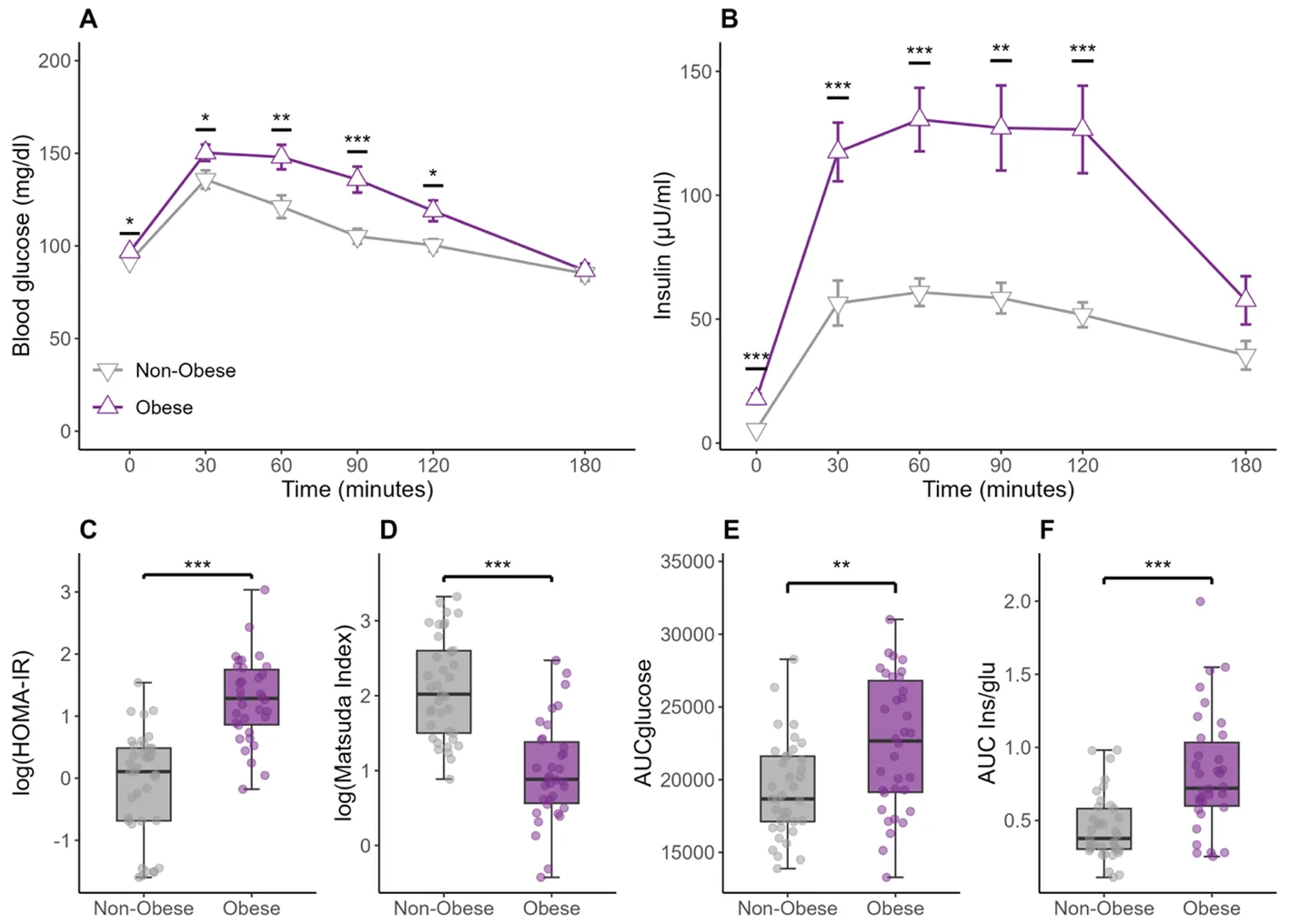
Glucose–insulin dynamics, insulin sensitivity, fasting insulin resistance, and OGTT-derived metabolic indices in women with PCOS according to obesity status. Plasma glucose **(A)** and insulin **(B)** concentrations during the 3-hour oral glucose tolerance test (OGTT), as well as log(HOMA-IR) **(C)**, log(Matsuda index) **(D)**, AUCglucose_(0-180)_ **(E)**, and the insulin-to-glucose AUC ratio (AUCins/AUCglu) **(F)** in non-obese (BMI <30 kg/m², grey) and obese (BMI ≥30 kg/m², purple) women with PCOS. Glucose and insulin concentrations are shown at 0, 30, 60, 90, 120, and 180 min after ingestion of 75 g oral glucose at 0 min. Time-course data are presented as means ± SEM; summary indices are box plots with individual data points. n = 37 (non-obese) and n = 34 (obese). *p < 0.05, **p < 0.005, and ***p < 0.0005 vs non-obese (unpaired t-tests, as appropriate).

During the 3-hour OGTT, obese women showed higher plasma glucose concentrations, particularly between 60 and 120 min (**Figure 1A**), and higher insulin concentrations throughout the OGTT than non-obese women (**Figure 1B**). Consistent with these profiles, obese women had higher fasting insulin resistance, as reflected by higher log(HOMA-IR) values (**Figure 1C**) and lower insulin sensitivity, as reflected by lower log(Matsuda index) values (**Figure 1D**). In addition, obese participants showed greater glycemic exposure during the OGTT, reflected by higher AUC glucose_(0–180)_ (**Figure 1E**), and a higher insulin response relative to glucose exposure, reflected by a higher AUCins/AUCglu ratio (**Figure 1F**).

### Exploratory pooled correlations suggest BMI as the strongest correlate of insulin sensitivity and insulin resistance indices

**Figure 2:**
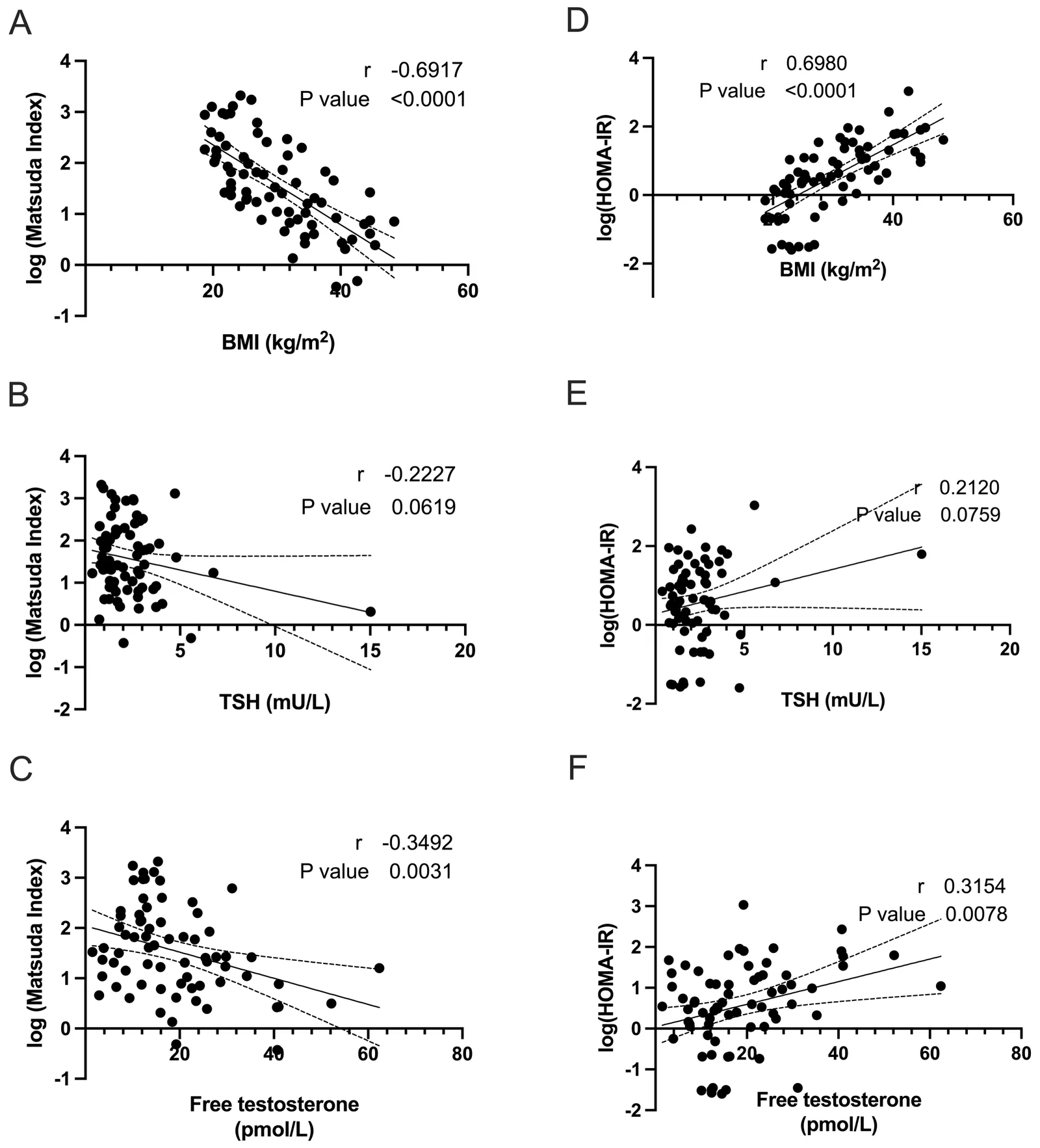
Exploratory associations of BMI, TSH, and free testosterone with log(Matsuda) and log(HOMA-IR) in women with PCOS. Scatterplots show exploratory associations of BMI **(A, D)**, TSH **(B, E)**, and free testosterone **(C, F)** with log-transformed Matsuda index **(A–C)** and log(HOMA-IR) **(D–F)**. The solid line indicates the fitted linear regression line and the dashed lines the 95% confidence interval. Pearson correlation coefficients and corresponding p-values are shown in each panel.

In pooled exploratory Pearson correlation analyses across participants, insulin sensitivity, as assessed by log(Matsuda), was inversely associated with BMI (**Figure 2A**), was not linked to TSH (**Figure 2B**), and was inversely associated with free testosterone (**Figure 2C**). A similar pattern was observed for insulin resistance, as assessed by log(HOMA-IR), which was positively associated with BMI (**Figure 2D**), was not associated with TSH (**Figure 2E**), and was positively associated with free testosterone (**Figure 2F**). This pattern was consistent with the univariable regression analyses (**Supplementary Table 8**), in which BMI was associated with log(Matsuda) (β = −0.0781, 95% CI −0.0977 to −0.0585; p < 0.0001), log(HOMA-IR) (β = 0.092, 95% CI 0.070 to 0.115; p < 0.0001), and AUCglucose_(0–180)_ (β = 211, 95% CI 90 to 332; p = 0.0009). Free testosterone was associated with log(Matsuda) (β = −0.0260, 95% CI −0.0429 to −0.0091; p = 0.0031) and log(HOMA-IR) (β = 0.028, 95% CI 0.008 to 0.048; p = 0.0078), but not clearly with AUCglucose_(0–180)_ (β = 83, 95% CI −2 to 169; p = 0.0543).

### Parsimonious multivariable models identify BMI as the only consistent independent predictor of insulin sensitivity, fasting insulin resistance, and glucose exposure

To assess independent associations with key metabolic outcomes while limiting model complexity, parsimonious multivariable linear regression models including BMI, age and total testosterone were fitted in 67 participants (**Table 2**). Across all three models, BMI was the only consistently significant independent predictor. Higher BMI was independently associated with lower insulin sensitivity (log[Matsuda]: β = −0.078, 95% CI −0.099 to −0.058; p < 0.0001), higher fasting insulin resistance (log[HOMA-IR]: β = 0.094, 95% CI 0.069 to 0.118; p < 0.0001), and greater glucose exposure during the OGTT (AUCglucose_(0–180)_: β = 188.8, 95% CI 60.2 to 317.4; p = 0.0047). Neither age nor total testosterone was independently associated with any of these outcomes. The findings were consistent in broader models additionally including TSH, androstenedione, and AMH. BMI remained the only consistently significant independent predictor, while the additional endocrine parameters were not independently associated with the metabolic outcomes (**Supplementary Tables 3**, **S4**).

**Table 2:** Parsimonious multivariable linear regression models for metabolic outcomes in women with PCOS.

Parameter	log(Matsuda) β [95% CI]	P-value	log(HOMA-IR) β [95% CI]	P-value	AUCglucose β [95% CI]	P-value
BMI (kg/m²)	-0.078 [-0.099; -0.058]	<0.0001	0.094 [0.069; 0.118]	<0.0001	188.8 [60.2; 317.4]	0.0047
Age (years)	0.009 [-0.014; 0.031]	0.4479	-0.008 [-0.034; 0.018]	0.5282	21.5 [-117.9; 160.8]	0.7591
Total testosterone (nmol/L)	-0.195 [-0.580; 0.189]	0.3132	0.029 [-0.422; 0.480]	0.8977	1267.6 [-1136.3; 3671.5]	0.2960

Values are unstandardized regression coefficients (β) with 95% confidence intervals (CI) from separate multivariable linear regression models for log-transformed Matsuda index, log-transformed HOMA-IR, and AUCglucose_(0–180)_. BMI, age, and directly measured total testosterone were entered simultaneously into each model. To permit direct comparison with the corresponding broader sensitivity models, all primary models were fitted in the same complete-case sample of 67 participants.

The Matsuda index and HOMA-IR were natural-log transformed before modeling. Accordingly, regression coefficients for these outcomes refer to changes on the logarithmic scale. A negative coefficient for log(Matsuda) indicates lower insulin sensitivity, whereas a positive coefficient for log(HOMA-IR) indicates higher fasting insulin resistance. A positive coefficient for AUCglucose_(0–180)_ indicates greater glucose exposure during the OGTT. All p-values are two-sided.

AUC, area under the curve; BMI, body mass index; CI, confidence interval; HOMA-IR, homeostatic model assessment of insulin resistance; OGTT, oral glucose tolerance test; PCOS, polycystic ovary syndrome.

The adjusted R² values were 0.471 for log(Matsuda), 0.473 for log(HOMA-IR), and 0.108 for AUCglucose_(0–180)_ (**Supplementary Table 6**). Diagnostic plots indicated an acceptable approximation of the linear-model assumptions for log(Matsuda) and log(HOMA-IR). In contrast, the AUCglucose models showed limited explanatory performance, suggested non-constant residual variance, and potentially influential observations (**Supplementary Figures 3–S6**). In an additional sensitivity analysis of the broader AUCglucose model, exclusion of two observations identified as potentially influential modestly increased the adjusted R² from 0.086 to 0.163. BMI remained independently associated with AUCglucose_(0–180)_ (β = 210, 95% CI 77 to 340; p = 0.0023), while the overall model fit remained limited (**Supplementary Table 7**).

### Sensitivity analysis using free testosterone instead of total testosterone

In sensitivity analyses replacing directly measured total testosterone with calculated free testosterone in otherwise identical parsimonious models (n = 66), the results were materially unchanged (**Supplementary Table 5**). BMI remained independently associated with log(Matsuda) (β = −0.076, 95% CI −0.097 to −0.054; p < 0.0001), log(HOMA-IR) (β = 0.090, 95% CI 0.065 to 0.116; p < 0.0001), and AUCglucose_(0–180)_ (β = 177.6, 95% CI 39.1 to 316.1; p = 0.0128). Calculated free testosterone was not independently associated with log(Matsuda) (β = −0.007, 95% CI −0.022 to 0.007; p = 0.3093), log(HOMA-IR) (β = 0.006, 95% CI −0.011 to 0.023; p = 0.4652), or AUCglucose_(0–180)_ (β = 33.4, 95% CI −57.6 to 124.5; p = 0.4656). The corresponding adjusted R² values were 0.484, 0.485, and 0.104, respectively, indicating comparable model fit when calculated free testosterone was substituted for total testosterone (**Supplementary Table 6**).

### Change-in-estimate analyses

Adding metformin use to the parsimonious main models changed the BMI coefficient by +0.5% for log(Matsuda), −1.1% for log(HOMA-IR), and +9.4% for AUCglucose_(0–180)_. Thus, all changes remained below 10%, although the change was more pronounced for AUCglucose_(0–180)_. Adding recorded cycle day attenuated the BMI coefficient by 6.7% for log(Matsuda), 5.8% for log(HOMA-IR), and 6.0% for AUCglucose_(0–180)_. BMI remained significantly associated with all three outcomes after inclusion of cycle day (all p ≤ 0.0065). The individual addition of progesterone, estradiol, LH, or FSH changed the BMI coefficient by no more than 1.6% for log(Matsuda), 2.0% for log(HOMA-IR), and 4.9% for AUCglucose_(0–180)_. Complete change-in-estimate results are provided in **Supplementary Table 9**.

## Discussion

In this retrospective analysis of women with PCOS, obese and non-obese participants differed strikingly in their metabolic phenotype, despite only limited differences in androgen-related parameters. Obese women showed higher glucose concentrations during the 3-hour OGTT, markedly higher insulin responses, lower insulin sensitivity, higher fasting insulin resistance, greater glucose exposure, and a higher insulin response relative to glucose exposure than non-obese women. In contrast, endocrine differences were less pronounced and were not supported by independent associations in multivariable models. This pattern remained unchanged in sensitivity analyses replacing total testosterone with free testosterone. BMI was the only consistent independent predictor of insulin sensitivity, fasting insulin resistance, and glucose exposure. Within this PCOS cohort, the metabolically adverse phenotype was more closely associated with BMI and obesity status than with the androgen-related features assessed, although comparisons with women without PCOS were not available.

These findings are in line with previous work suggesting that body weight may be more informative than Rotterdam phenotype for defining metabolic status in women with PCOS, as the Rotterdam classification mainly reflects differences in ovulatory function and androgen secretion but does not adequately capture the metabolic dimension of the syndrome (1). A similar pattern has been reported in other cohorts, in which obesity was associated with a less favorable metabolic profile in women with PCOS (15). In addition, a large cross-sectional study suggested that PCOS alone may not sufficiently explain the presence of metabolic syndrome once abdominal obesity is taken into account (16). Consistent with this, the most pronounced separation in our cohort was metabolic rather than endocrine, and multivariable analyses indicated that metabolic dysfunction was more closely linked to BMI than to androgen-related parameters. Together, these findings suggest that obesity status and BMI may be particularly relevant for metabolic risk stratification in PCOS.

Metformin use was more frequent among obese participants and may reflect treatment by indication, as women with a more adverse metabolic phenotype are more likely to receive metformin. Adding metformin use to the parsimonious main models changed the BMI coefficient only minimally for log(Matsuda) (+0.5%) and log(HOMA-IR) (−1.1%), whereas the change was more pronounced for AUCglucose_(0–180)_ (+9.4%). Residual confounding related to treatment duration, dosage, and adherence cannot be excluded.

Previous studies have likewise placed insulin resistance at the center of metabolic dysfunction in PCOS. A narrative review on the long-term metabolic consequences of PCOS identified insulin resistance as a key metabolic feature of the syndrome and noted that it may already be present in lean women, but is considerably more frequent in obesity, affecting approximately 30% of lean and 70% of obese women with PCOS (5). Additionally, rising BMI seems to aggravate insulin resistance more strongly in women with PCOS than in women without PCOS (8). PCOS is increasingly recognized as a condition characterized not only by chronic anovulation and androgen excess, but also by substantial metabolic and cardiometabolic abnormalities (17). A systematic review and meta-analysis of euglycaemic-hyperinsulinaemic clamp studies demonstrated a reduction in insulin sensitivity of 27% in women with PCOS compared with controls, independent of BMI, while a higher BMI further exacerbated this reduction by 15% (8). Overall, these data suggest that insulin resistance in PCOS is not simply a secondary correlate of hyperandrogenism, but rather a central metabolic abnormality whose clinical expression becomes more pronounced in the presence of excess weight.

In our cohort, the impact of obesity was not only reflected by higher fasting insulin resistance, but became even more apparent during dynamic glucose challenge, where obese women showed less favorable glucose-insulin responses throughout the OGTT. A cross-sectional study likewise reported that dynamic insulin resistance, rather than hyperandrogenism, tracked with increasing menstrual dysfunction in PCOS (11), supporting the idea that dynamic metabolic phenotyping may capture clinically relevant heterogeneity within the syndrome more effectively than static endocrine characteristics alone.

A study of adrenal hyperandrogenism in women with PCOS showed that adrenal hyperandrogenism was not associated with more severe insulin resistance or a worse lipid profile despite higher androgen levels (18). More generally, high testosterone and low SHBG concentrations have been associated with insulin resistance, whereas adrenal hyperandrogenemia does not appear to worsen insulin resistance to the same extent (8, 19). This distinction is particularly relevant in the context of obesity, because obesity and insulin resistance may reduce hepatic SHBG production and thereby increase free testosterone levels (20). Accordingly, higher free testosterone in women with PCOS may partly reflect altered androgen bioavailability secondary to metabolic dysfunction rather than a generalized androgen-driven phenotype. This interpretation is consistent with our data, as DHEA-S, androstenedione, and total testosterone were similar between obese and non-obese women, whereas free and bioavailable testosterone were higher in obese women. Moreover, calculated free testosterone was not independently associated with the metabolic outcomes in the parsimonious sensitivity models. The obese group was on average 3.7 years older than the non-obese group. Although age was included as a covariate in the multivariable models of metabolic outcomes, this difference remains relevant when interpreting the endocrine group comparisons. In particular, the lower estradiol and AMH concentrations observed in obese women may at least partly reflect age-related differences rather than obesity or metabolic dysfunction per se. Taken together, these findings suggest that free and bioavailable testosterone may reflect altered androgen bioavailability in the context of obesity and insulin resistance, rather than acting as independent drivers of metabolic dysfunction.

The clinical relevance of this phenotype is supported by long-term outcome data. Women with PCOS have a higher prevalence of impaired glucose tolerance, type 2 diabetes, and metabolic syndrome, including in BMI-matched analyses (4). In addition, prospective data identified BMI, fasting glucose, and glucose area under the curve as predictors of incident type 2 diabetes, whereas higher SHBG was associated with lower risk (6). This is particularly relevant to our findings, as glucose exposure during the OGTT was higher in obese women and remained independently associated with BMI, although the limited explanatory performance of the AUCglucose models warrants caution. Beyond glycaemic traits, a prospective study in young women with PCOS linked increased carotid intima-media thickness and reduced flow-mediated dilation more closely to insulin resistance and lower adiponectin than to BMI itself (21). Thus, while BMI was the strongest clinical correlate of metabolic dysfunction in our cohort, the underlying metabolic heterogeneity is likely more complex than body mass alone.

Adiposity alone is unlikely to fully explain metabolic heterogeneity in PCOS. A recent systematic review highlighted that adipose tissue dysfunction, more than excess adiposity per se, may contribute to the metabolic and inflammatory abnormalities of PCOS (9). Although our study cannot distinguish adipose mass from adipose dysfunction, BMI remained the strongest and most consistent correlate of metabolic dysfunction in our cohort. This suggests that BMI and obesity status are readily available clinical markers of metabolic risk in this setting, even if the underlying biology is more complex than body mass alone. From a clinical perspective, these findings suggest that metabolic phenotyping in PCOS should focus more strongly on obesity status, BMI, and dynamic markers of glucose–insulin handling when the question is cardiometabolic risk. This does not diminish the diagnostic relevance of hyperandrogenism, but indicates that endocrine classification and metabolic risk stratification are not interchangeable. This distinction is particularly relevant given the heterogeneity of PCOS (22–24), its long-term cardiometabolic burden (25, 26), and its broader health care burden (27).

Several limitations should be acknowledged. First, the retrospective cross-sectional design precludes causal inference. Second, the sample size was moderate, and the cohort was derived from a tertiary referral center, which may limit generalizability and increase the risk of referral-related selection effects (28, 29). Third, because the study did not include a control group without PCOS, the findings should be interpreted as reflecting metabolic heterogeneity within PCOS. The present data cannot determine whether the association between BMI and metabolic dysfunction is specific to PCOS or primarily reflects the well-established metabolic consequences of obesity in the general population. Comparative studies including BMI-matched women with and without PCOS are required to address whether PCOS modifies the metabolic effects of adiposity. Fourth, direct measures of insulin sensitivity, body fat distribution, adipose tissue function, and inflammatory biomarkers were not available. BMI is an imperfect surrogate of adiposity and does not capture visceral or ectopic fat burden. Measures such as waist circumference, waist-to-hip ratio, or direct assessment of visceral adiposity may provide additional insight into metabolic risk. Although multivariable models were adjusted for key covariates, residual confounding cannot be excluded, particularly in view of age differences and differences in medication use between groups. Recorded cycle day modestly attenuated the BMI estimates across all three metabolic outcomes, although BMI remained significantly associated with each outcome (**Supplementary Table 9**). However, recorded cycle day may not reliably reflect physiological cycle phase in women with oligo- or amenorrhea. In addition, the multivariable models were based on complete-case analyses, which reduced the effective sample size and may have introduced selection bias. Moreover, while model fit was acceptable for log(Matsuda) and log(HOMA-IR), the AUCglucose models explained only a limited proportion of outcome variability and showed potentially influential observations in the diagnostic plots. Exclusion of two such observations modestly improved model fit without materially changing the association with BMI; nevertheless, the AUCglucose findings should be interpreted with caution.

Finally, although most androgen-related markers were not independently associated with metabolic dysfunction in our models, these findings should not be interpreted as evidence against a contributory role of hyperandrogenism. Circulating androgen concentrations may not fully reflect functionally relevant androgen exposure at the tissue level because of interindividual differences in androgen receptor sensitivity and peripheral androgen metabolism.

In conclusion, our findings support a more adverse metabolic phenotype in obese compared with non-obese women with PCOS, with insulin resistance as a central feature. Obese women with PCOS showed a clearly more adverse glucose–insulin profile than non-obese women, and BMI was the only consistent independent predictor of insulin sensitivity, fasting insulin resistance, and glucose exposure. Most androgen-related markers were not independently associated with metabolic dysfunction, and the higher free and bioavailable testosterone concentrations observed in obese women may reflect altered androgen bioavailability rather than generalized androgen excess. Overall, these data indicate that within this PCOS cohort, the metabolically adverse phenotype was more closely associated with BMI and obesity status than with the androgen-related features assessed.

## Data Availability

The original contributions presented in the study are included in the article/**Supplementary Material**. Further inquiries can be directed to the corresponding author upon reasonable request.
