# Supplementary material for: Metabolic dysfunction in women with PCOS is more closely linked to BMI than to hyperandrogenism
Source: Front Endocrinol (Lausanne). 2026 Aug 31;17:1923923. doi: 10.3389/fendo.2026.1923923 (PMC13572115; doi:10.3389/fendo.2026.1923923)
Supplement: Supplementary file 1 [file SupplementaryFile1.pdf]

Supplementary Methods (Naderi et al. Metabolic dysfunction in women with PCOS is more closely linked to BMI than to hyperandrogenism)

Laboratory assessment

Venous blood samples were obtained under fasting conditions after a 12-hour overnight fast as part of routine clinical work-up. Laboratory parameters included plasma glucose (mg/dL), glycated hemoglobin (HbA1c; %), insulin (µU/mL), C-peptide (ng/mL), thyroid-stimulating hormone (TSH; mU/L), free thyroxine (fT4; pmol/L), luteinizing hormone (LH; IU/L), follicle-stimulating hormone (FSH; IU/L), prolactin (ng/mL), progesterone (ng/mL), estradiol (pg/mL), total testosterone (nmol/L), sex hormone-binding globulin (SHBG; nmol/L), dehydroepiandrosterone sulfate (DHEA-S; µg/dL), androstenedione (ng/mL), 17-hydroxyprogesterone (17-OH-progesterone; ng/dL), and anti-Müllerian hormone (AMH; µg/L). The LH/FSH ratio was calculated. Free and bioavailable testosterone were calculated according to the Vermeulen formula and reported in pmol/L and nmol/L, respectively. HbA1c was determined in EDTA blood samples using the Tosoh Automated Glycohemoglobin Analyzer HLC-723G8 (Tosoh Bioscience, Tokyo, Japan) by high-performance liquid chromatography. Glucose was determined in NaF plasma using the Atellica CH GluH_3 assay (Siemens Healthcare Diagnostics, Erlangen, Germany) based on the hexokinase method. TSH and fT4 were measured in serum using automated chemiluminescence immunoassays on the ADVIA Centaur platform (Bayer Vital, Fernwald, Germany). LH, FSH, prolactin, progesterone, estradiol, total testosterone, DHEA-S, and androstenedione were measured using chemiluminescence-based immunoassays on the ADVIA Centaur platform (Bayer Vital, Fernwald, Germany). SHBG was measured by immunometric assay on the IMMULITE 2000 XPi platform (Siemens Healthcare Diagnostics, Erlangen, Germany). Insulin was measured on the Atellica IM Analyzer using the Atellica IM Insulin (IRI) assay (Siemens Healthineers, Erlangen, Germany), and C-peptide was measured on the IMMULITE 2000 using the IMMULITE 2000 C-Peptide assay (Siemens Healthcare Diagnostics, Erlangen, Germany). 17-hydroxyprogesterone (17-OH-progesterone) was analyzed in an external laboratory (Labor Eberhard, Dortmund, Germany) according to the laboratory's routine procedures.

Samples for AMH measurement were refrigerated after receipt in the local laboratory and transported on weekdays once daily under cooled conditions to an external accredited laboratory (Labor Limbach, Heidelberg, Germany). AMH was measured using Roche Elecsys AMH assays on cobas immunoassay platforms; assay versions changed during the study period according to routine laboratory implementation. All laboratory investigations, except for AMH and 17-OH-progesterone, were performed in the Division of Laboratory Medicine, University Hospital Essen. Except for samples sent to the external laboratories, all samples were transferred to the local laboratory immediately after collection and analyzed on the day of collection.

Oral glucose tolerance test and derived indices

All participants underwent a standardized 75-g OGTT after a 12-hour overnight fast. Following collection of fasting blood samples at baseline (0 min), participants ingested 75 g of oral glucose. Venous blood samples were then collected at 30, 60, 90, 120 and 180 minutes for measurement of plasma glucose, serum insulin, and C-peptide. Glucose concentrations are reported in mg/dL, insulin in µU/mL, and C-peptide in ng/mL. The Matsuda index (ISI(comp)) is an index of whole-body insulin sensitivity derived from the simultaneous assessment of insulin and glucose levels obtained at 0, 30, 60, 90, and 120 minutes of the OGTT.

$$\mathrm{ISI(comp)} = \frac{10000}{\sqrt{g_0 \times i_0 \times \frac{(g_0\cdot 15+g_{30}\cdot 30+g_{60}\cdot 30+g_{90}\cdot 30+g_{120}\cdot 15}{120} \times \frac{(i_0\cdot 15+i_{30}\cdot 30+i_{60}\cdot 30+i_{90}\cdot 30+i_{120}\cdot 15}{120}}}$$

g = glucose, i = insulin, 0 = fasting; 30, 60, 90, 120 minutes after a glucose challenge with 75 g glucose. This index represents a composite of both hepatic and peripheral tissue sensitivity to insulin and considers insulin sensitivity in the basal state and after the ingestion of a defined glucose load [1]. It is strongly correlated ($r = 0.73$, $p < 0.0001$) with the hyperinsulinemic-euglycemic clamp, which is a direct measure of insulin sensitivity. The Matsuda index has been shown to be superior to the HOMA-IR in detecting risk and reclassifying insulin resistance [2]. It has thus been suggested as a suitable surrogate measure for the hyperinsulinemic-euglycemic clamp [1], which is not feasible for routine clinical application.

Fasting insulin resistance was assessed using the homeostatic model assessment of insulin resistance (HOMA-IR), calculated as [3]: HOMA-IR = (fasting insulin × fasting glucose) / 405

Glycaemic exposure during the OGTT was quantified as the area under the glucose concentration-time curve from 0 to 180 minutes [AUC $\text{glucose}_{(0\text{–}180\ \text{min})}$] and expressed as mg/dL·min. Insulin exposure was quantified analogously as AUC $\text{insulin}_{(0\text{–}180\ \text{min})}$ and expressed as µU/mL·min, and C-peptide exposure as AUC $\text{C-peptide}_{(0\text{–}180\ \text{min})}$ and expressed as ng/mL·min. These AUCs were calculated by the trapezoidal rule using measurements obtained at 0, 30, 60, 90, 120 and 180 minutes. Relative insulin response to glucose exposure was assessed as the ratio of AUC $\text{insulin}_{(0\text{–}180\ \text{min})}$ to AUC $\text{glucose}_{(0\text{–}180\ \text{min})}$ [AUCins/AUCglu].

Statistical analysis

Participants were analyzed according to obesity status (BMI <30.0 vs ≥30.0 kg/m²). Continuous variables are presented as mean ± standard deviation (SD) when approximately normally distributed and as median with interquartile range (IQR) when distributional assumptions were not met. Categorical variables are presented as absolute counts and percentages. Missing data were limited to AMH in four participants, SHBG in one participant, which also prevented calculation of free and bioavailable testosterone, DHEA-S in one participant, and C-peptide in one participant. Analyses were performed

using all available observations unless complete-case analysis was required, and the corresponding sample sizes are reported in the tables.

Between-group comparisons were performed using unpaired Student's t-tests or Mann–Whitney U tests for continuous variables and chi-square tests or Fisher's exact tests for categorical variables, as appropriate. The overall distribution of the four menstrual-disturbance grades was compared between obesity groups using a global chi-square test. Exploratory comparisons of metabolic outcomes according to clinical or phenotypic hyperandrogenism were performed using two-sided Mann–Whitney U tests for Ferriman–Gallwey score <8 versus ≥ 8, presence versus absence of Ludwig-classified alopecia, and hyperandrogenic Rotterdam phenotypes A–C versus phenotype D.

Exploratory Pearson correlation analyses were performed in the pooled cohort to assess associations of BMI, TSH, AMH, and calculated free testosterone with log-transformed Matsuda index and log-transformed HOMA-IR. Univariable linear regression models were additionally fitted for candidate predictors using log(Matsuda), log(HOMA-IR), and AUCglucose(0–180) as dependent variables. All available observations were used for each predictor–outcome combination.

Multivariable linear regression models were used to assess independent associations with metabolic dysfunction. Because of their right-skewed distributions and to improve fulfillment of the linear-model assumptions, the Matsuda index and HOMA-IR were natural-log transformed before modeling.

Based on the a priori study hypothesis addressing the relative contributions of adiposity and biochemical androgen status, the primary parsimonious models included BMI, age, and directly measured total testosterone as simultaneous predictors of log(Matsuda), log(HOMA-IR), and AUCglucose(0–180). These predictors were selected to represent adiposity, a relevant demographic covariate, and the primary directly measured biochemical androgen marker. To permit direct comparison with the broader total-testosterone models, the parsimonious models were fitted in the same complete-case sample of 67 participants.

Broader multivariable models additionally including TSH, androstenedione, and AMH were retained as sensitivity analyses to determine whether reduction of the number of predictors materially affected the parameter estimates. In separate sensitivity analyses, directly measured total testosterone was replaced by calculated free testosterone in the corresponding parsimonious and broader models. Models including calculated free testosterone were fitted in 66 participants because SHBG, which is required for its calculation, was unavailable in one participant.

Change-in-estimate analyses were performed by adding potential confounders individually to the parsimonious main models including BMI, age, and directly measured total testosterone. The percentage change in the BMI coefficient relative to the corresponding parsimonious main model was calculated.

Model assumptions and potentially influential observations were evaluated using residuals-versus-fitted, normal Q–Q, scale-location, and residuals-versus-leverage plots, including Cook's distance. Model fit was summarized using the adjusted coefficient of determination (adjusted R^2). For AUCglucose(0–180), an additional sensitivity analysis of the broader total-testosterone model was performed after excluding two observations identified as potentially influential in the diagnostic plots.

Statistical analyses and graphical visualization were performed using R, version 4.5.0. (R Core Team 2025; R: A Language and Environment for Statistical Computing. R Foundation for Statistical Computing, Vienna, Austria. https://www.R-project.org/) and GraphPad Prism, version 11.0.0 (93) (GraphPad Software, San Diego, CA, USA). All tests were two-sided, and a p-value <0.05 was considered statistically significant.

Figure S1. Study flow diagram.

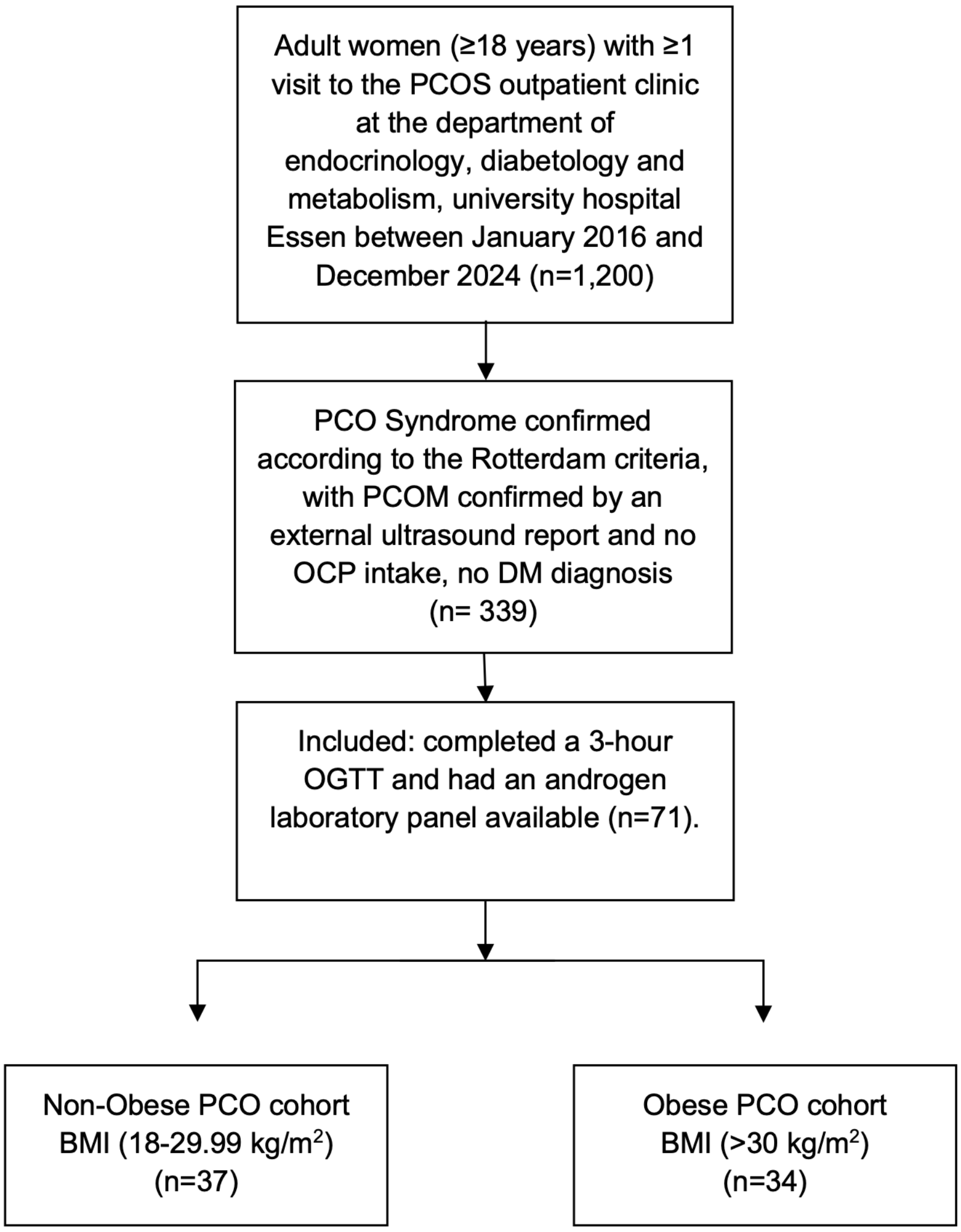

Figure S2. Distribution of LH, FSH, progesterone, and estradiol concentrations across recorded cycle-day categories in women with PCOS.

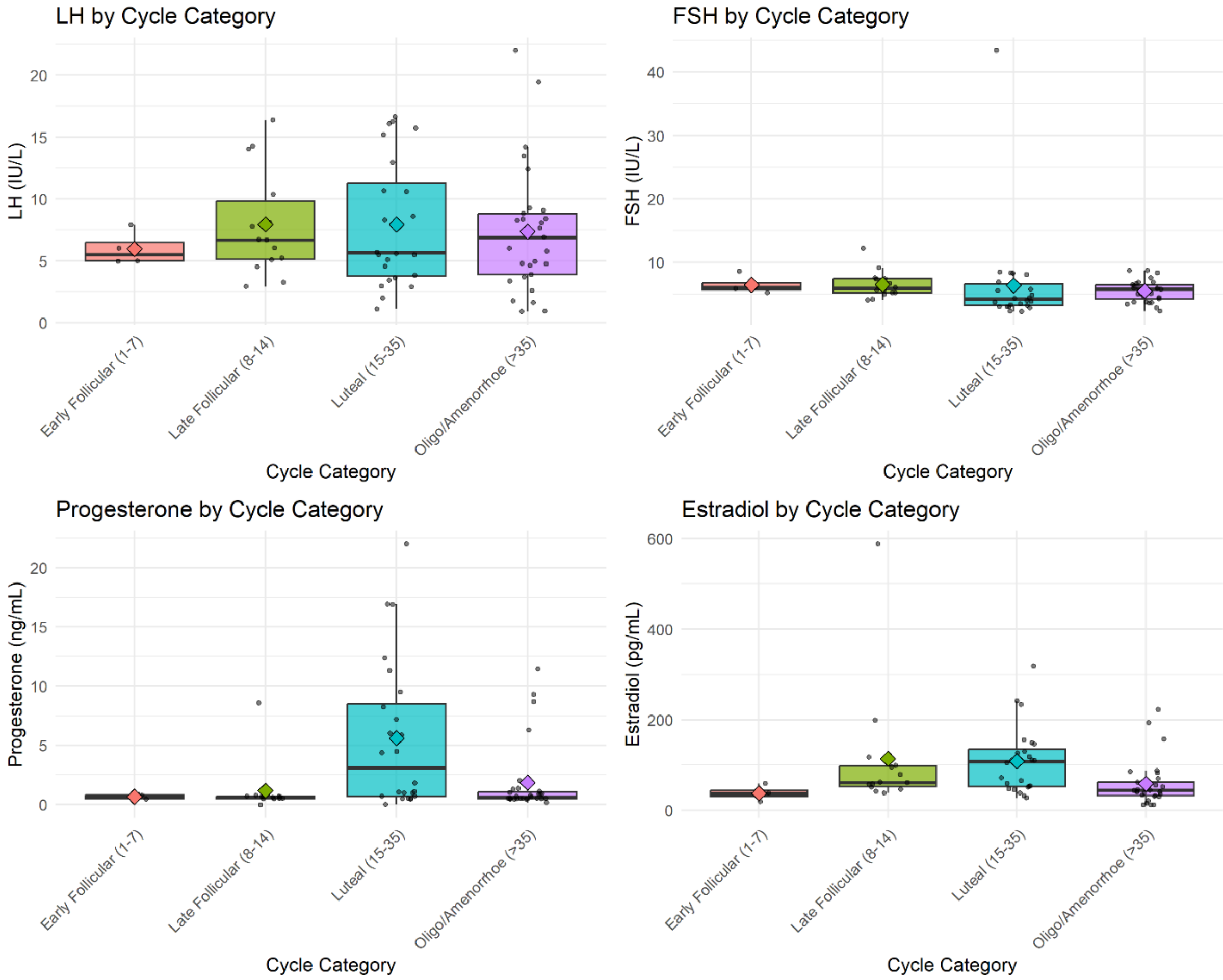

Recorded cycle-day categories were defined as early follicular phase (days 1–7), late follicular phase (days 8–14), luteal phase (days 15–35), and oligo-/amenorrhea (>35 days). Data are shown descriptively; no formal statistical comparisons were performed.

Figure S3. Model diagnostic plots for the primary parsimonious multivariable regression models using total testosterone.

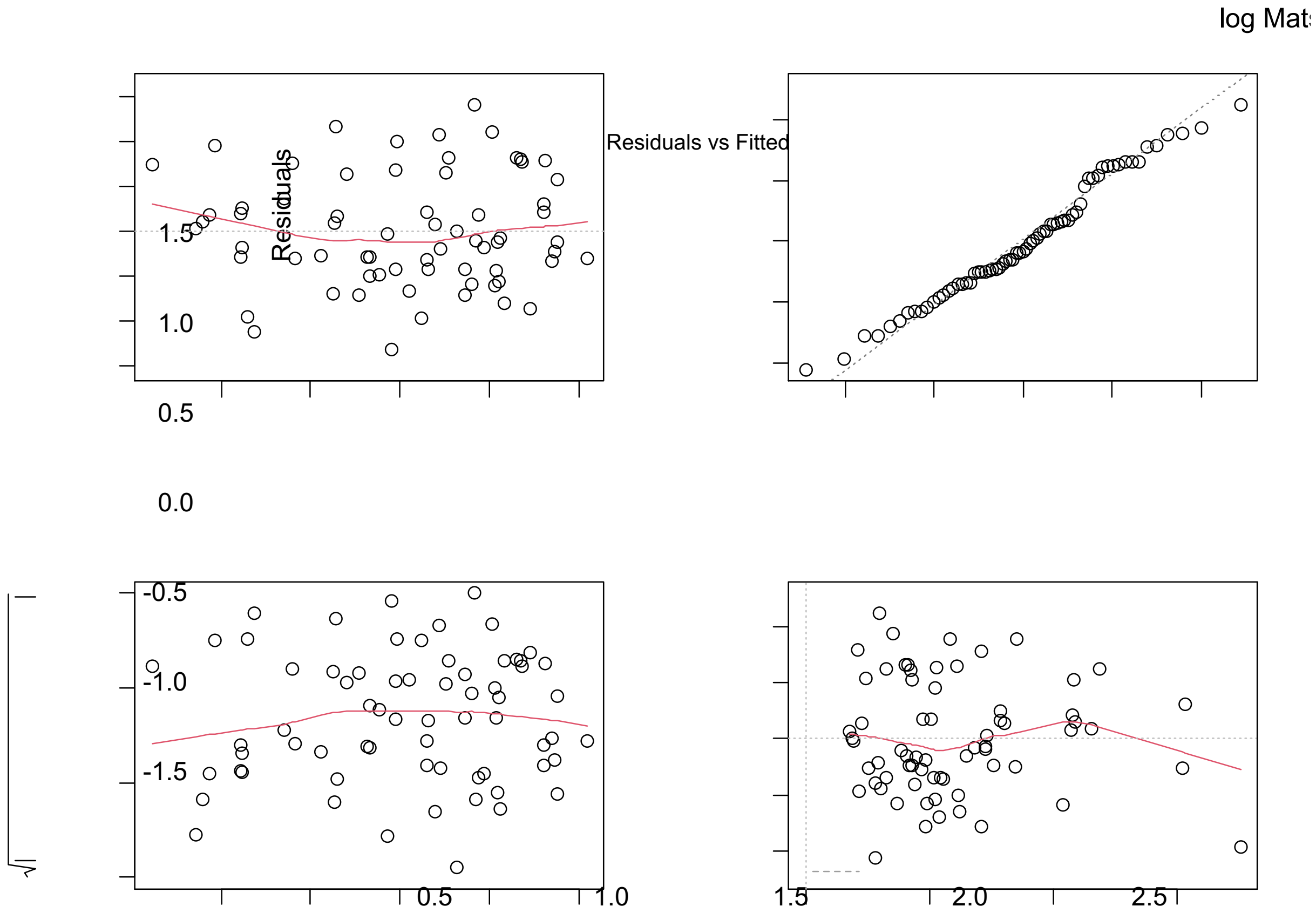

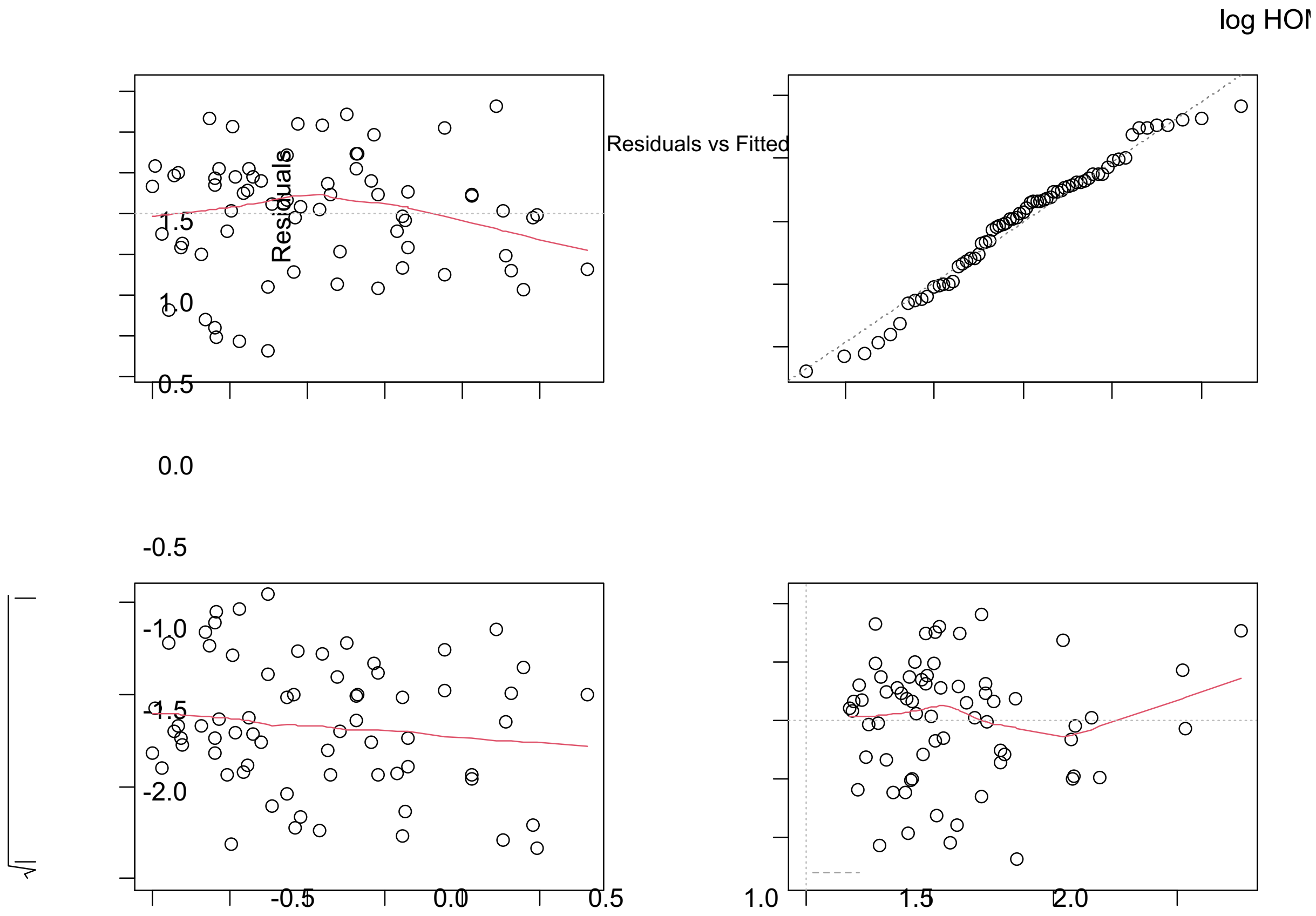
log HOI
Residuals vs Fitted
Residuals
1.5
1.0
0.5
0.0
-0.5
-1.0
-1.5
-2.0
-0.5
0.0
0.5
1.0
1.5
2.0

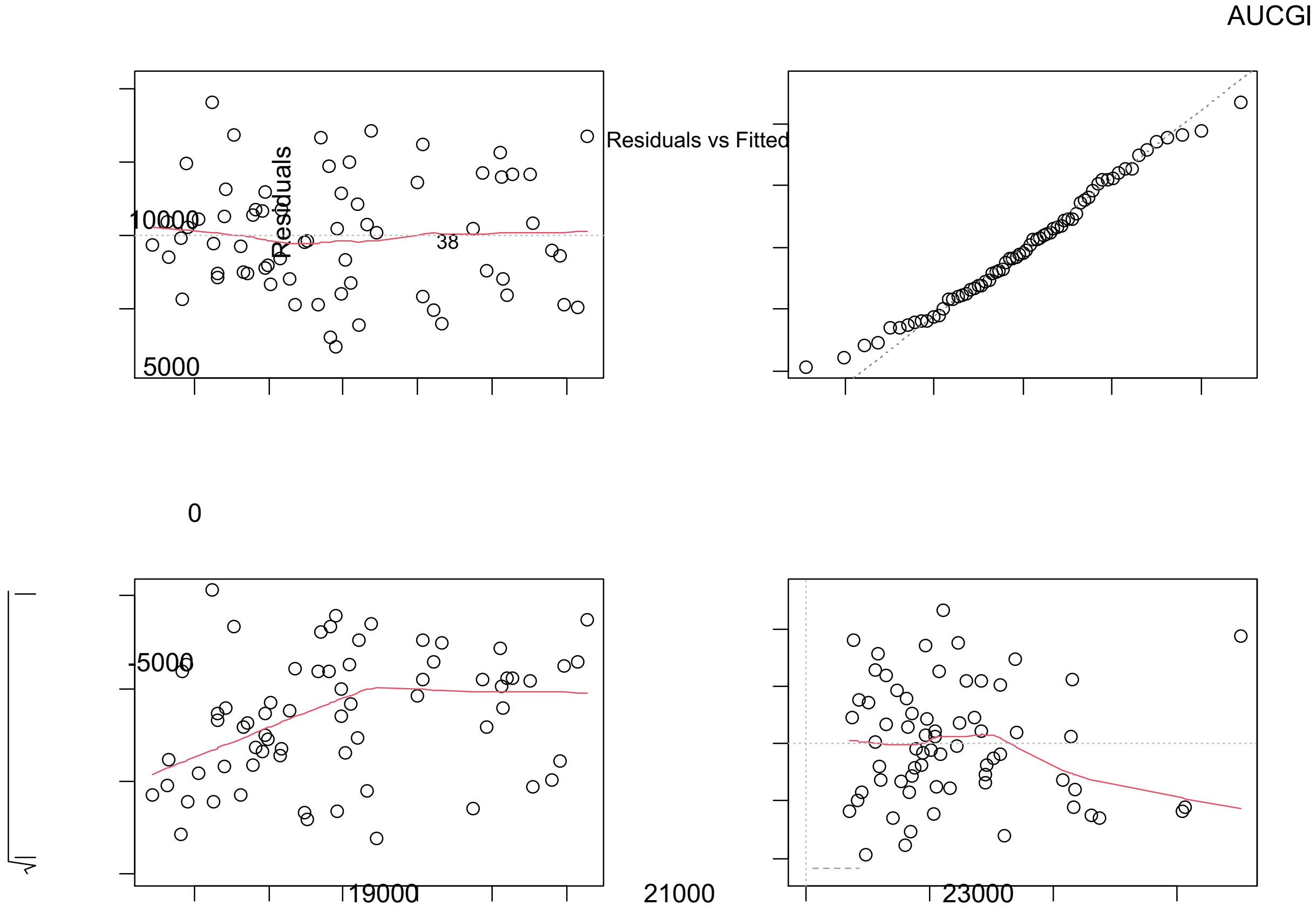

Diagnostic plots are shown for the models with log(Matsuda), log(HOMA-IR), and AUCglucose(0–180) as dependent variables. For each outcome, residuals-versus-fitted, normal Q–Q, scale-location, and residuals-versus-leverage plots with Cook's distance are presented.

Figure S4. Model diagnostic plots for the parsimonious sensitivity models using calculated free testosterone.

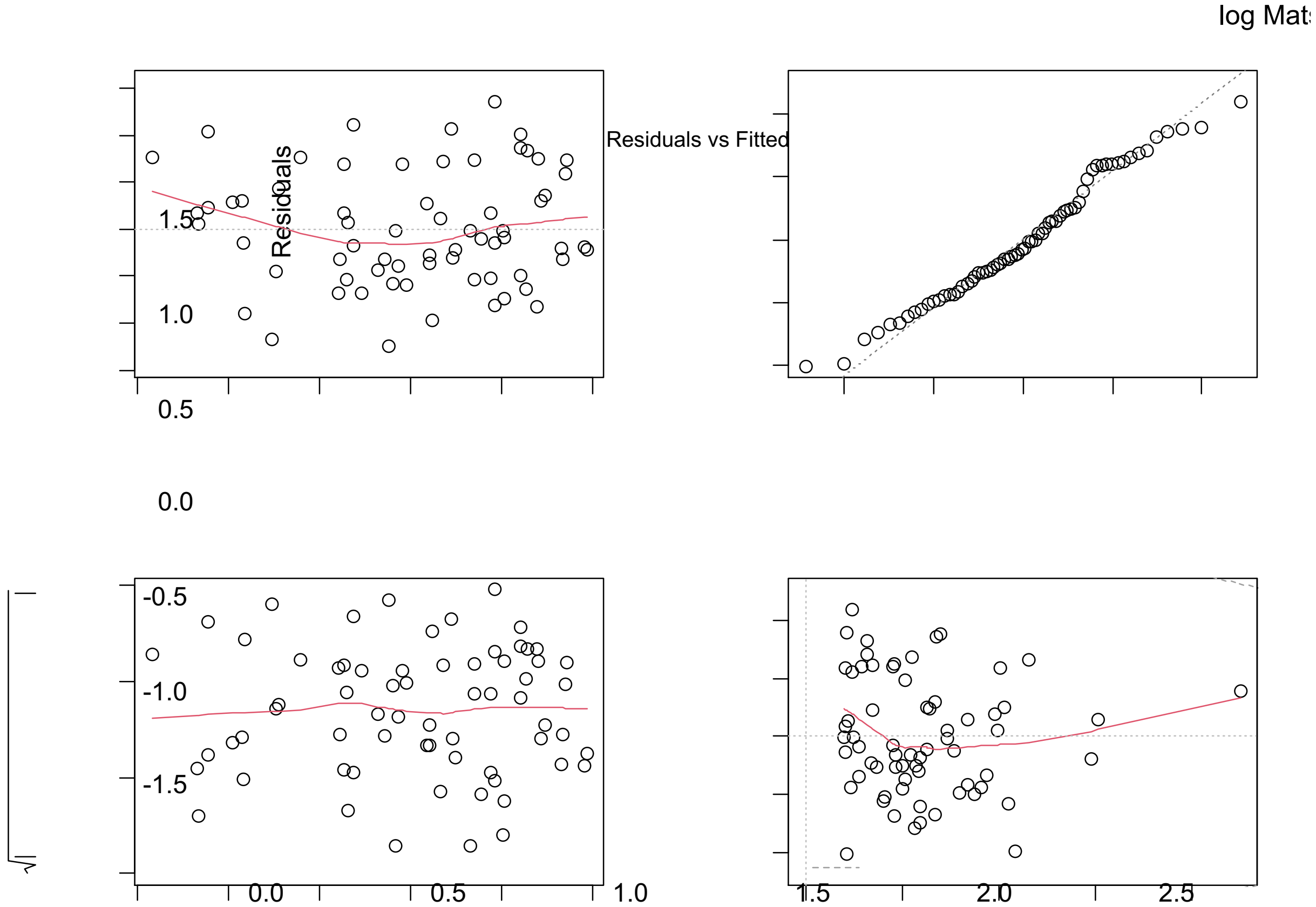

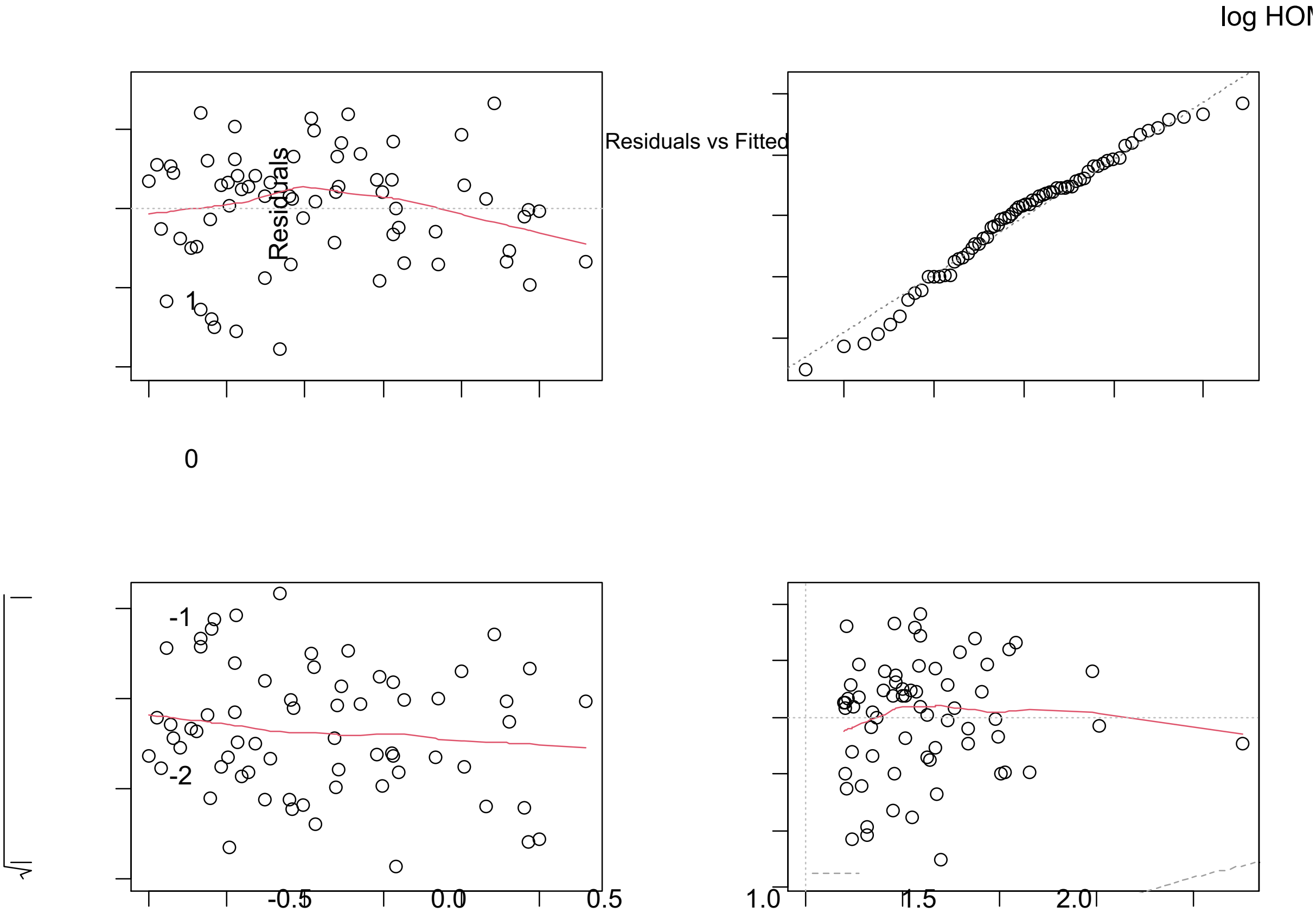
log HOI
Residuals vs Fitted
Residuals
1
0
-1
-2
-0.5
0.0
0.5
1.0
1.5
2.0

AUCGl

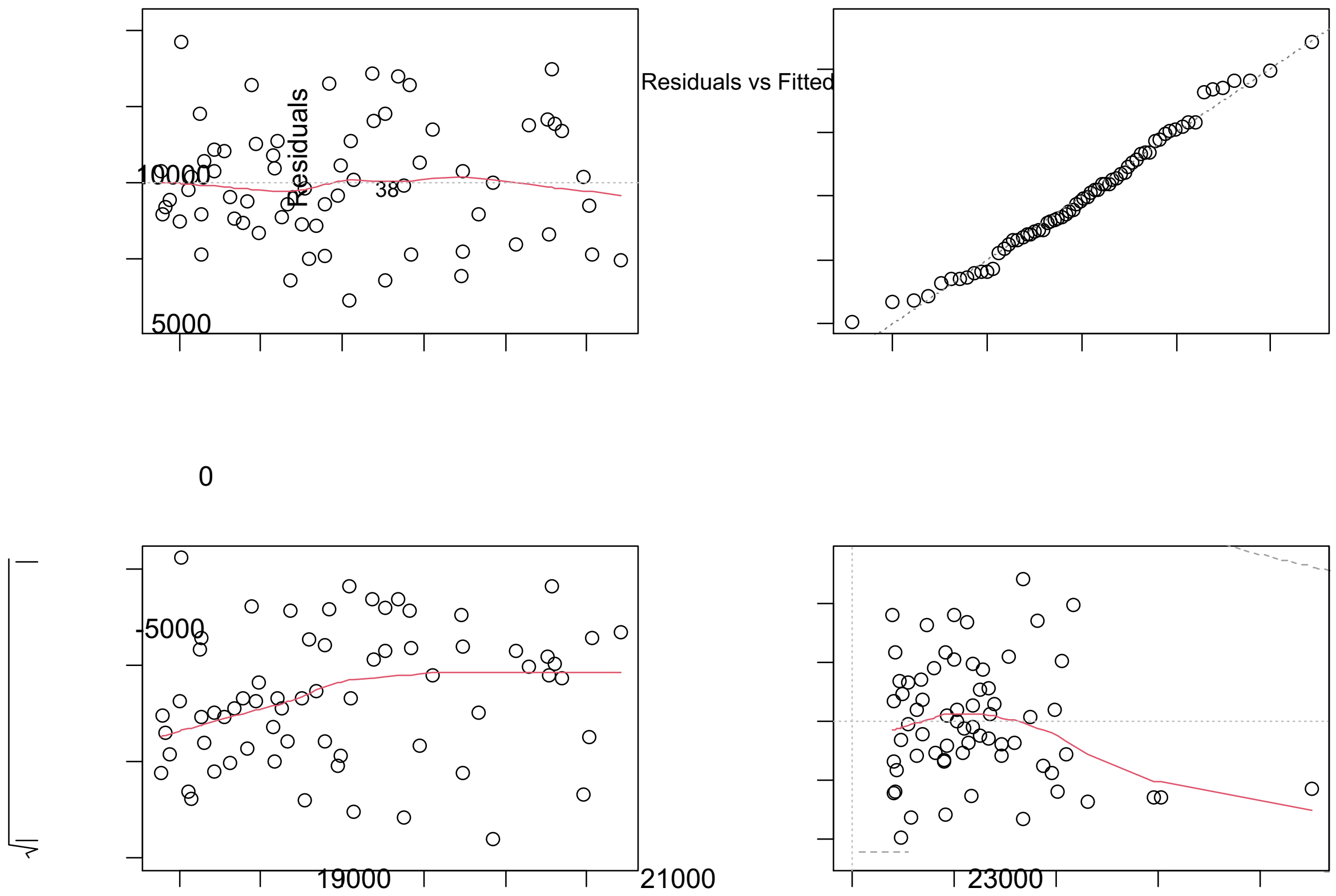

Diagnostic plots are shown for the models with log(Matsuda), log(HOMA-IR), and AUCglucose(0–180) as dependent variables. For each outcome, residuals-versus-fitted, normal Q–Q, scale-location, and residuals-versus-leverage plots with Cook's distance are presented.

Figure S5. Model diagnostic plots for the broader multivariable regression models using total testosterone.

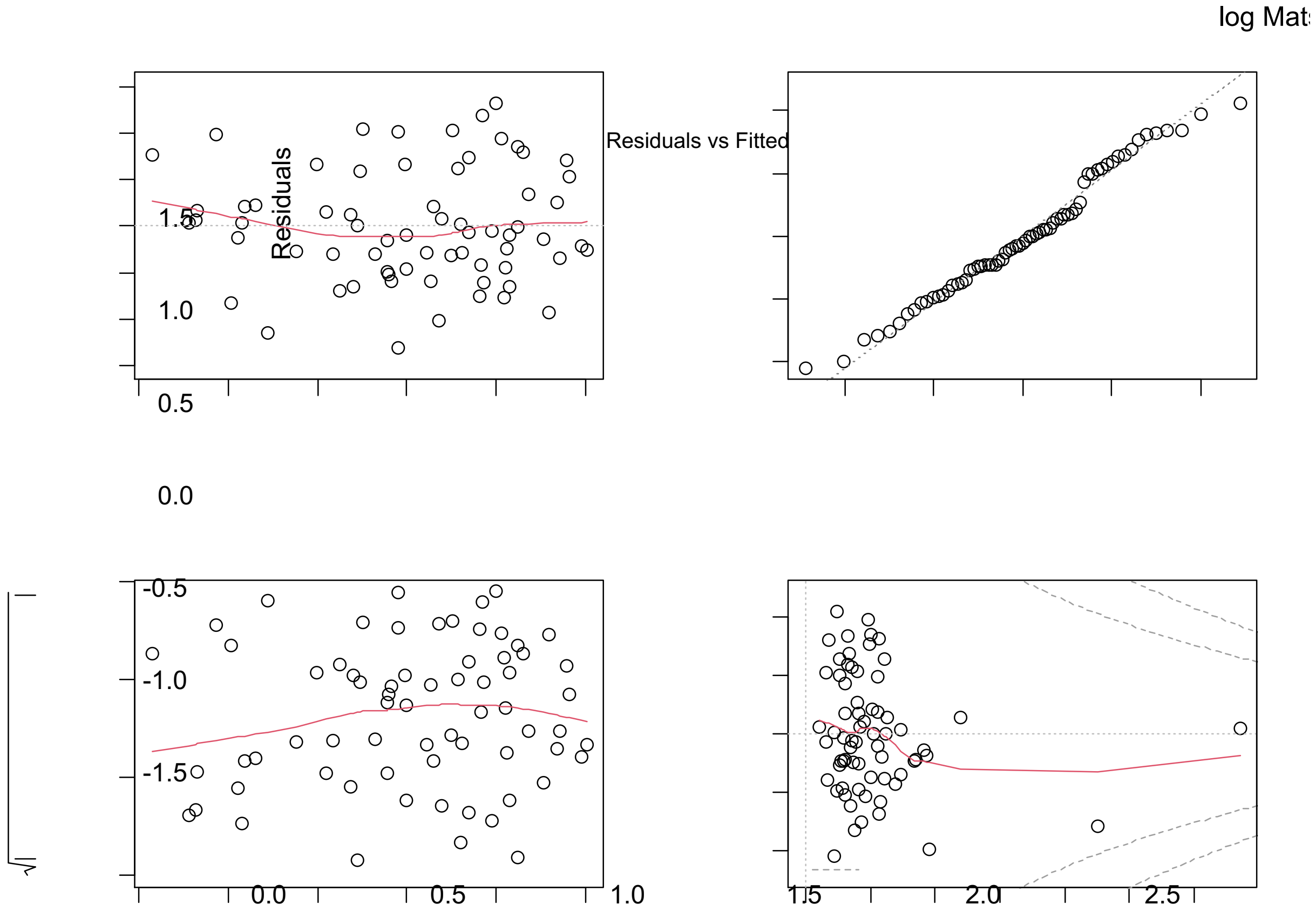

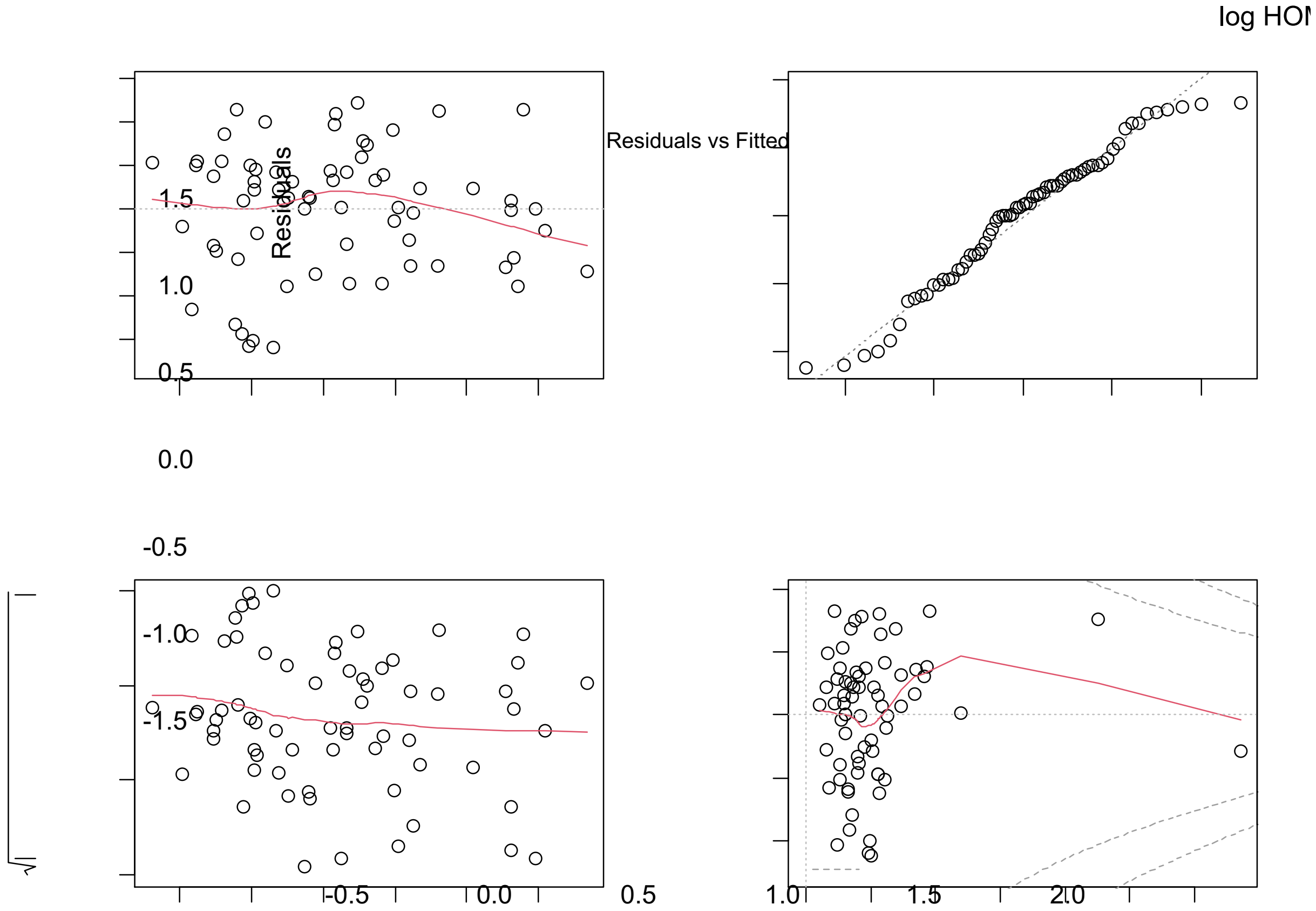

log HOI
Residuals vs Fitted
Residuals
1.5
1.0
0.5
0.0
-0.5
-1.0
-1.5
-0.5
0.0
0.5
1.0
1.5
2.0

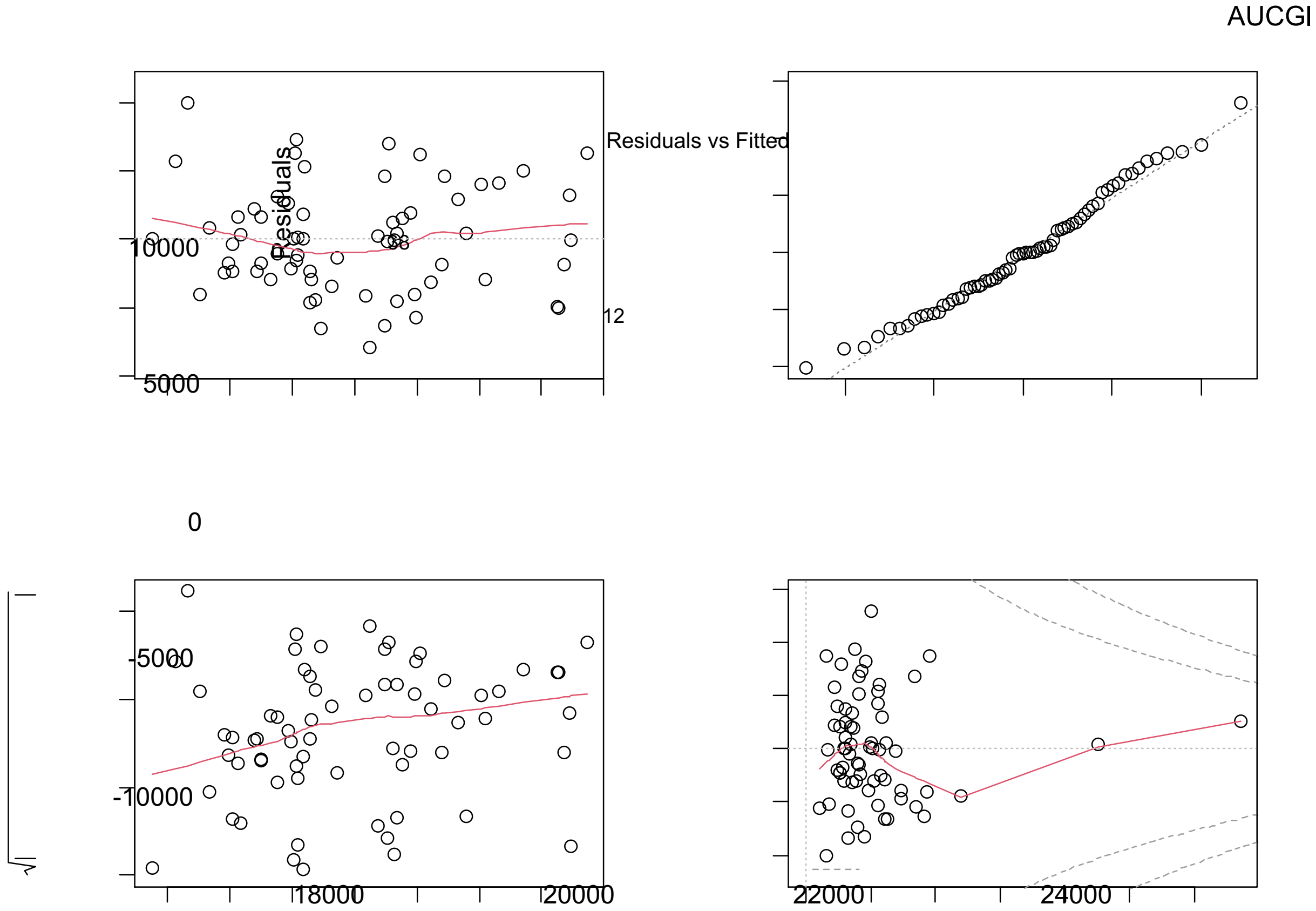

Diagnostic plots are shown for the models with log(Matsuda), log(HOMA-IR), and AUCglucose(0–180) as dependent variables. For each outcome, residuals-versus-fitted, normal Q–Q, scale-location, and residuals-versus-leverage plots with Cook's distance are presented.

Figure S6. Model diagnostic plots for the broader sensitivity models using calculated free testosterone.

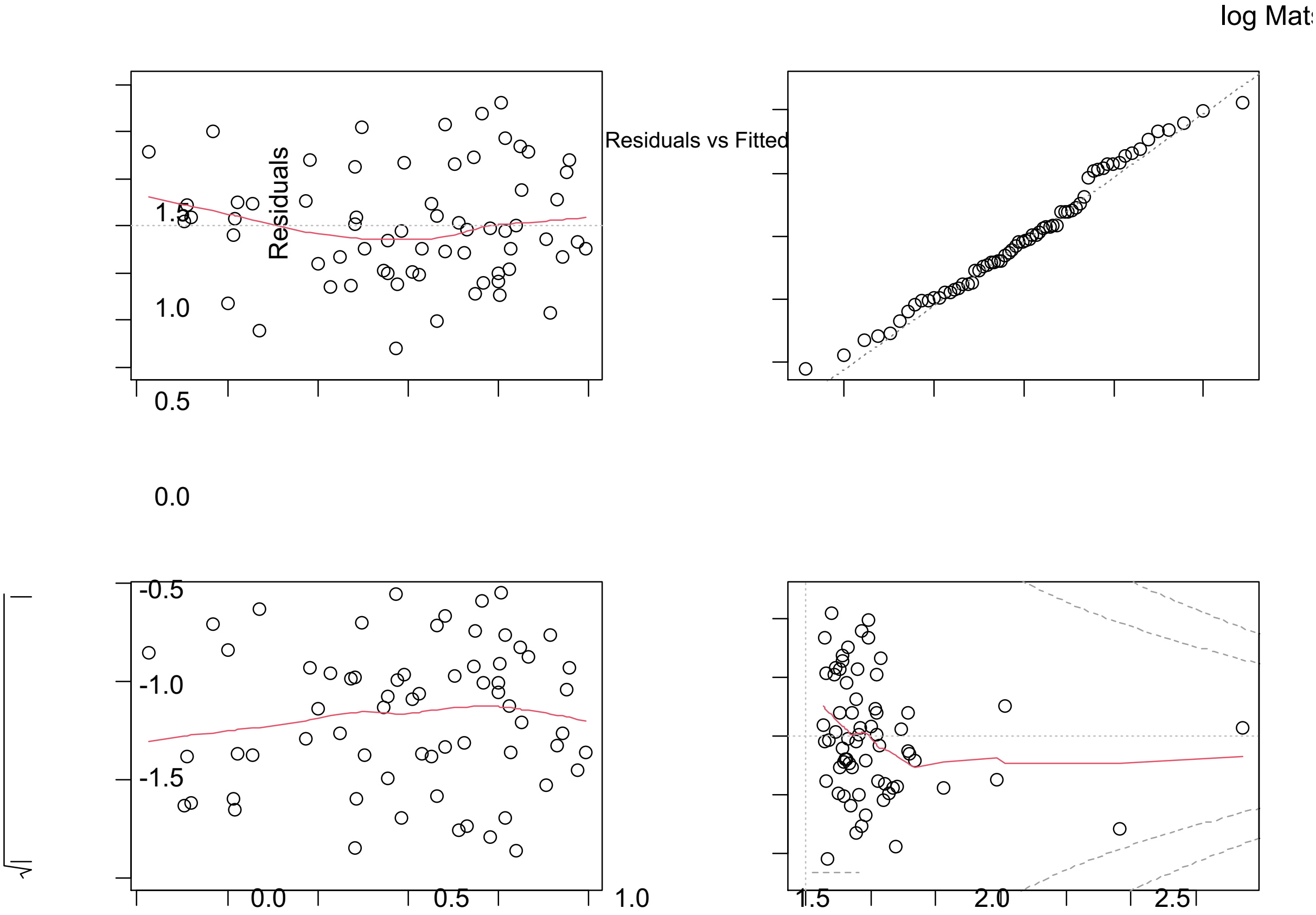

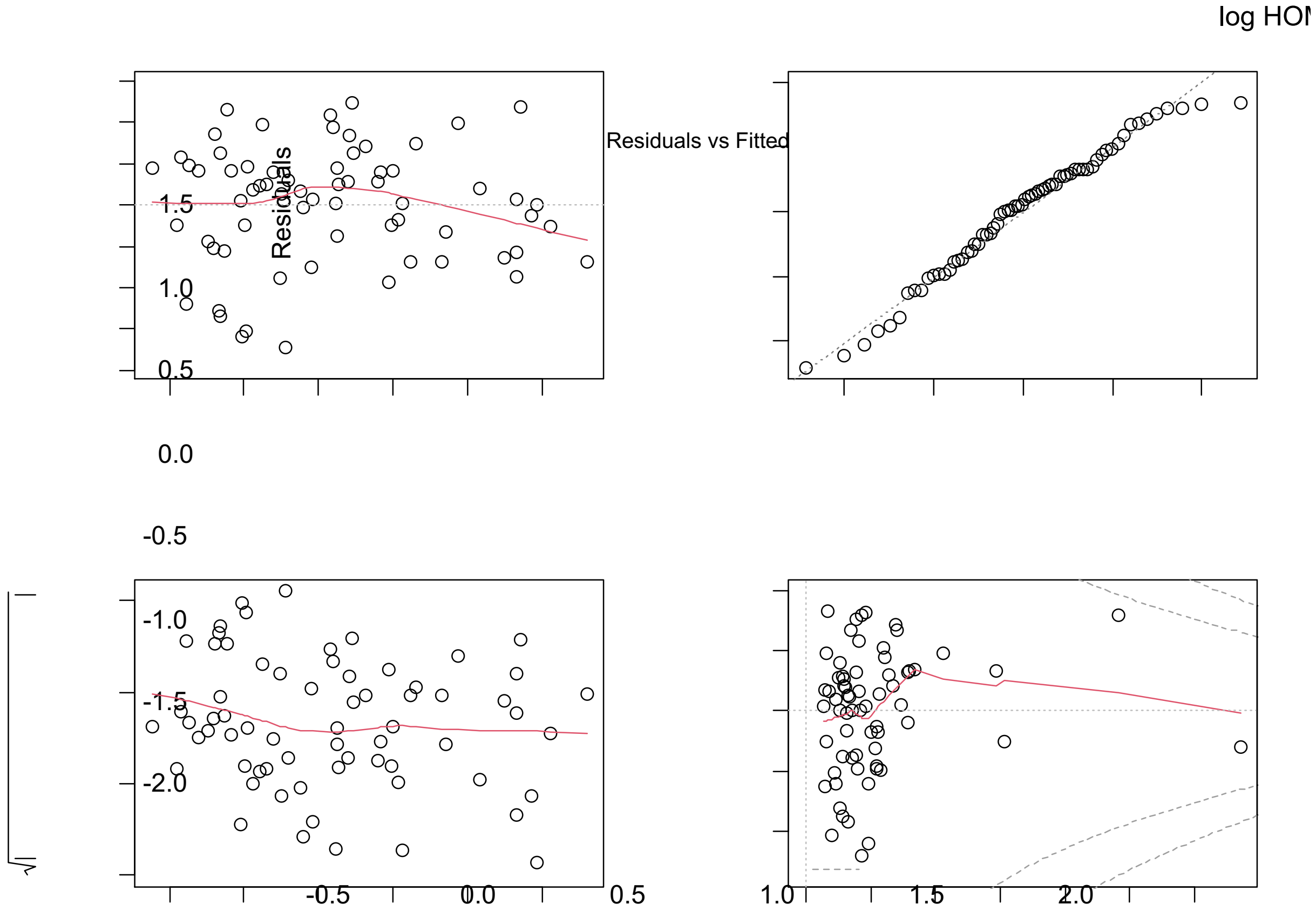
log HOI
Residuals vs Fitted
Residuals
1.5
1.0
0.5
0.0
-0.5
-1.0
-1.5
-2.0
-0.5
0.0
0.5
1.0
1.5
2.0

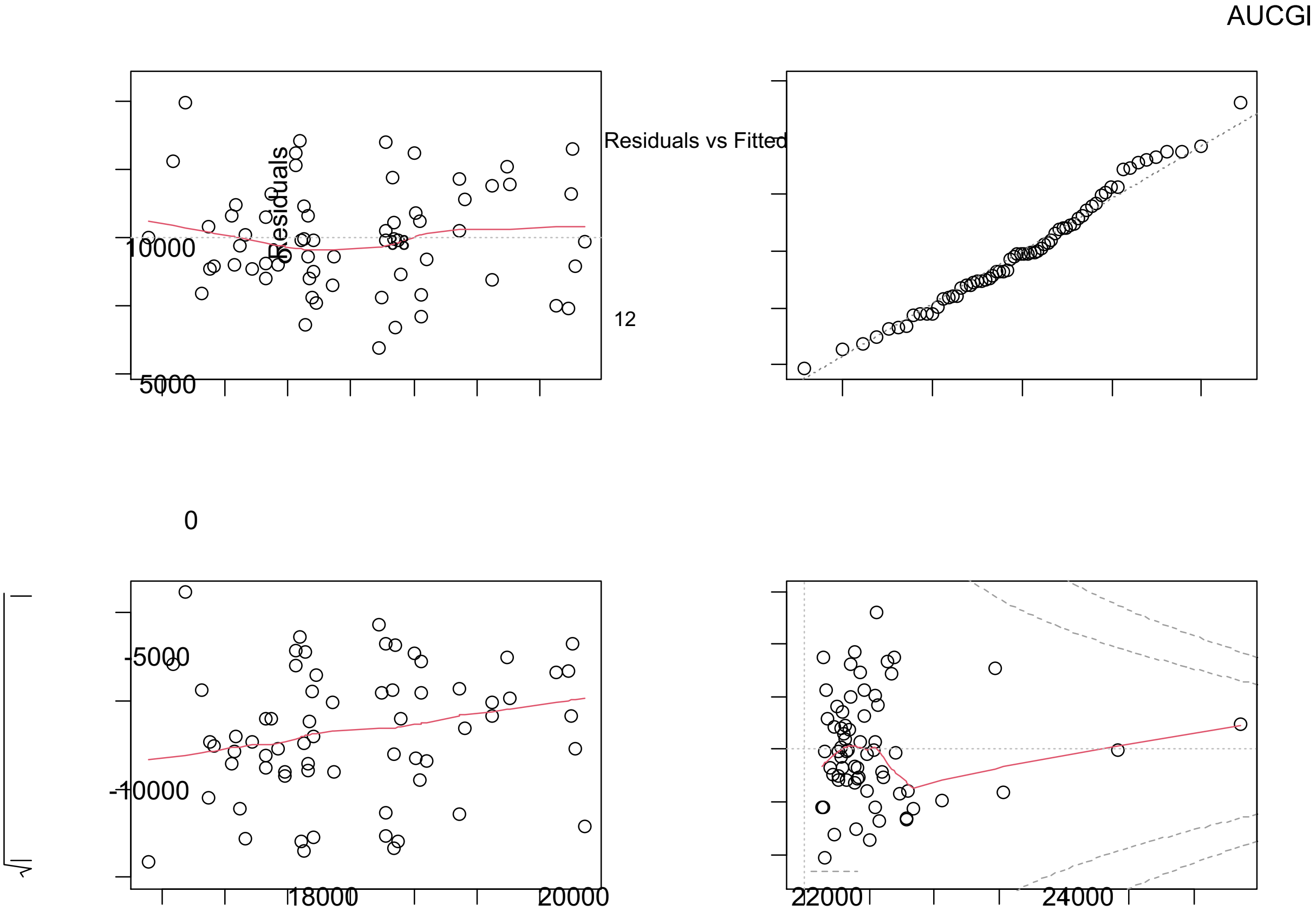

Diagnostic plots are shown for the models with log(Matsuda), log(HOMA-IR), and AUCglucose(0–180) as dependent variables. For each outcome, residuals-versus-fitted, normal Q–Q, scale-location, and residuals-versus-leverage plots with Cook’s distance are presented.

Table S1. Clinical and reproductive characteristics and medication use in women with PCOS stratified by obesity status (non-obese, BMI <30 kg/m²; obese, BMI ≥30 kg/m²).

Variable	Non-Obese	Obese
n	37	34
PCOM (yes/no) [%]	29/8 [78/22]	29/5 [85/15]
Ferriman-Gallwey score (<8/8-16/17-25/>25) [%]	16/13 /2/6 [43/35/5/16]	8/14/6/6 [24/41/18/18]
Ludwig-classified alopecia (yes/no) [%]	4/33 [11/89]	3/31 [9/91]
PCOS phenotype (A/B/C/D) [%]	26/7/0/4 [70/19/0/11]	24/4/0/6 [71/12/0/18]
Metformin use, n [%]	9 [24]	15 [44]
Levothyroxine use, n [%]	6 [16]	14 [41]
Beta-blocker use, n [%]	2 [5]	4 [12]
Menstrual disturbance grade (0/1/2/3) [%]	13/15/5/4 [35/41/14/11]	10/10/5/9 [29/29/14/26]

Table S2. Exploratory comparisons of metabolic outcomes according to clinical and phenotypic markers of hyperandrogenism

Parameter	Group	n	log(Matsuda index), median (IQR)	p-value	log(HOMA-IR), median (IQR)	p-value	AUCglucose, median (IQR)	p-value
Ferriman-Gallwey score	<8	24	1.82 (0.95-2.67)		0.31 (-0.57-1.22)		20115 (17213-24263)	
	≥8	47	1.41 (0.85-2.14)	0.185	0.639 (0.11-1.31)	0.166	20145 (17295-23790)	0.940
Ludwig-classified alopecia	Absent	64	1.47 (0.89-2.22)		0.60 (0.05-1.30)		20100 (17224-23813)	
	Present	7	1.42 (0.50-2.95)	0.962	0.52 (-0.69-1.80)	0.974	20715 (17295-22815)	0.930
Rotterdam phenotype	A+B+C	61	1.50 (0.88-2.28)		0.54 (-0.17-1.29)		20145 (17310-23243)	
	D	10	1.34 (0.89-1.88)	0.478	0.88 (0.14-1.32)	0.465	19778 (16665-25399)	0.944

Data are presented as median (interquartile range). Exploratory comparisons were performed using two-sided Mann–Whitney U tests. Clinical hirsutism was defined as a Ferriman–Gallwey score ≥8. Hyperandrogenic Rotterdam phenotypes comprised phenotypes A–C, whereas phenotype D was classified as non-hyperandrogenic. HOMA-IR and the Matsuda index are presented after logarithmic transformation. These analyses were exploratory, and no adjustment for multiple testing was performed.

Table S3. Broader multivariable linear regression models for metabolic outcomes in women with PCOS using total testosterone.

Parameter	log Matsuda β [95% CI]	p-value	log HOMA-IR β [95% CI]	p-value (HOMA)	AUCglucose β [95% CI]	p-value
BMI (kg/m²)	-0.076 [-0.097; -0.054]	<0.0001	0.089 [0.064; 0.115]	<0.0001	195.8 [59.3; 332.3]	0.0057
Age (years)	0.005 [-0.019; 0.029]	0.6846	-0.007 [-0.036; 0.021]	0.6096	39.8 [-110.9; 190.6]	0.5990
TSH (mU/L)	-0.033 [-0.117; 0.050]	0.4279	0.046 [-0.053; 0.144]	0.3559	-164.3 [-688.2; 359.6]	0.5329
Total testosterone (nmol/L)	-0.028 [-0.530; 0.474]	0.9112	-0.069 [-0.658; 0.521]	0.8168	478.0 [-2663.1; 3619.0]	0.7619
Androstenedione (ng/mL)	-0.066 [-0.209; 0.077]	0.3568	0.038 [-0.130; 0.206]	0.6489	374.6 [-520.3; 1269.5]	0.4057
AMH (µg/L)	-0.001 [-0.054; 0.052]	0.9773	-0.017 [-0.079; 0.045]	0.5916	102.1 [-228.5; 432.6]	0.5390

Values are regression coefficients (β) with 95% confidence intervals from multivariable linear regression models. Separate models were fitted for log-transformed Matsuda index, log-transformed HOMA-IR, and $AUCglucose_{(0-180)}$. All listed predictors (BMI, age, TSH, total testosterone, androstenedione, and AMH) were entered simultaneously into each model.

Table S4. Sensitivity analysis using broader multivariable models with calculated free testosterone.

Parameter	log Matsuda β [95% CI]	p-value	log HOMA-IR β [95% CI]	p-value (HOMA)	AUCglucose β [95% CI]	p-value
BMI (kg/m²)	-0.075 [-0.098; -0.052]	<0.0001	0.087 [0.060; 0.114]	<0.0001	197.8 [52.9; 342.8]	0.0083
Age (years)	0.004 [-0.020; 0.028]	0.7690	-0.005 [-0.034; 0.023]	0.7129	42.4 [-109.9; 194.7]	0.5794
TSH (mU/L)	-0.036 [-0.118; 0.045]	0.3778	0.047 [-0.050; 0.144]	0.3384	-146.5 [-666.2; 373.1]	0.5747
Free testosterone (pmol/L)	-0.004 [-0.021; 0.013]	0.6764	0.007 [-0.014; 0.027]	0.5085	1.3 [-107.0; 109.7]	0.9803
Androstenedione (ng/mL)	-0.052 [-0.184; 0.079]	0.4301	-0.005 [-0.160; 0.151]	0.9536	433.1 [-401.4; 1267.5]	0.3033
AMH (µg/L)	-0.005 [-0.057; 0.047]	0.8376	-0.014 [-0.076; 0.048]	0.6502	125.6 [-205.3; 456.6]	0.4505

Table S5. Sensitivity analysis using parsimonious models with calculated free testosterone.

Parameter	log(Matsuda) β [95% CI]	p-value (Matsuda)	log(HOMA-IR) β [95% CI]	p-value (HOMA)	AUCglucose β [95% CI]	p-value (AUC)
BMI (kg/m²)	-0.076 [-0.097; -0.054]	<0.0001	0.090 [0.065; 0.116]	<0.0001	177.6 [39.1; 316.1]	0.0128
Age (years)	0.008 [-0.014; 0.030]	0.4840	-0.006 [-0.032; 0.021]	0.6702	18.7 [-122.6; 159.9]	0.7923
Free testosterone (pmol/L)	-0.007 [-0.022; 0.007]	0.3093	0.006 [-0.011; 0.023]	0.4652	33.4 [-57.6; 124.5]	0.4656

Table S6. Adjusted R² values for multivariable linear regression models

Model	log(Matsuda)	log(HOMA-IR)	AUCglucose
Broader model (total testosterone)	0.458	0.459	0.086
Broader model (free testosterone)	0.473	0.468	0.088
Parsimonious model (total testosterone)	0.471	0.473	0.108
Parsimonious model (free testosterone, sensitivity)	0.484	0.485	0.104

Table S7. Additional sensitivity analysis of the broader total-testosterone model for AUCglucose(0–180) after exclusion of two potentially influential observations.

Parameter	AUCglucose β [95% CI]	p-value (AUC)
BMI (kg/m²)	206.8 [77.0; 336.6]	0.0023
Age (years)	58.0 [-89.8; 205.9]	0.4353
TSH (mU/L)	-664.0 [-1516.8; 188.9]	0.1246
Total testosterone (nmol/L)	72.2 [-2956.0; 3100.3]	0.9621
Androstenedione (ng/mL)	648.7 [-225.1; 1522.5]	0.1427
AMH (µg/L)	106.8 [-206.6; 420.2]	0.4978

The adjusted R^2 of the model was 0.163.

Table S8. Univariable linear regression models for candidate predictors.
Pooled linear regression coefficients (β) with 95% confidence intervals (CI) and two-sided p values for each predictor and each outcome. Univariable regression models include the listed predictor only.

8.1 Univariable regression models for candidate predictors (log(Matsuda) as outcome)

Variable	β [95% CI]	p-value
BMI (kg/m²)	-0.0781 [-0.0977, -0.0585]	<0.0001
Age at OGTT (years)	-0.0101 [-0.0383, 0.0181]	0.4755
TSH (mU/L)	-0.1007 [-0.2065, 0.0052]	0.0619
Total testosterone (nmol/L)	-0.3349 [-0.8324, 0.1627]	0.1838
Free testosterone (pmol/L)	-0.0260 [-0.0429, -0.0091]	0.0031
Androstenedione (ng/mL)	-0.1336 [-0.2711, 0.0039]	0.0566
AMH (µg/L)	0.0329 [-0.0321, 0.0979]	0.3163
Cycle day at OGTT	-0.0083 [-0.0124, -0.0042]	0.0001
Severity of menstrual disorder	-0.3827 [-0.8774, 0.1121]	0.1273
Metformin use	-0.2618 [-0.7009, 0.1773]	0.2383
Thyroid hormone therapy	-0.2517 [-0.7142, 0.2109]	0.2815
Beta-blocker use	-0.4281 [-1.1754, 0.3192]	0.2571
Progesterone	0.0137 [-0.0320, 0.0594]	0.5508
17-OH-Progesterone	-0.0002 [-0.0023, 0.0020]	0.8662
Estradiol	0.0018 [-0.0006, 0.0043]	0.1349
LH	0.0290 [-0.0150, 0.0730]	0.1927
FSH	0.0290 [-0.0134, 0.0715]	0.1770
LH/FSH ratio	0.0762 [-0.1816, 0.3339]	0.5574
SHBG	0.0005 [-0.0020, 0.0030]	0.7055
DHEA-S	-0.0011 [-0.0028, 0.0005]	0.1739
HbA1c (%)	-0.7300 [-1.3528, -0.1073]	0.0223
Ferriman-Gallwey score	-0.0819 [-0.2797, 0.1159]	0.4116

8.2 Univariable regression models for candidate predictors (log(HOMA-IR) as outcome)

Variable	β [95% CI]	p-value
BMI (kg/m²)	0.0925 [0.0697, 0.1153]	<0.0001
Age at OGTT (years)	0.0165 [-0.0165, 0.0495]	0.3221
TSH (mU/L)	0.1125 [-0.0120, 0.2370]	0.0759
Total testosterone (nmol/L)	0.1861 [-0.4037, 0.7759]	0.5311
Free testosterone (pmol/L)	0.0277 [0.0075, 0.0479]	0.0078
Androstenedione (ng/mL)	0.0964 [-0.0677, 0.2604]	0.2453
AMH (μg/L)	-0.0606 [-0.1361, 0.0149]	0.1140
Cycle day at OGTT	0.0083 [0.0033, 0.0132]	0.0015
Severity of menstrual disorder	0.4789 [-0.1039, 1.0617]	0.1057
Metformin use	0.4519 [-0.0572, 0.9611]	0.0810
Thyroid hormone therapy	0.3370 [-0.2045, 0.8784]	0.2186
Beta-blocker use	0.3665 [-0.5145, 1.2475]	0.4095
Progesterone	-0.0031 [-0.0569, 0.0506]	0.9082
17-OH-Progesterone	0.0000 [-0.0025, 0.0025]	0.9969
Estradiol	-0.0021 [-0.0049, 0.0008]	0.1511
LH	-0.0365 [-0.0880, 0.0150]	0.1623
FSH	-0.0255 [-0.0756, 0.0247]	0.3143
LH/FSH ratio	-0.1334 [-0.4349, 0.1682]	0.3807
SHBG	-0.0008 [-0.0038, 0.0021]	0.5684
DHEA-S	0.0010 [-0.0010, 0.0030]	0.3159
HbA1c (%)	0.8566 [0.1258, 1.5875]	0.0223
Ferriman-Gallwey score	0.1235 [-0.1079, 0.3549]	0.2907

8.3 Univariable regression models for candidate predictors (AUCglucose$_{(0-180)}$ as outcome)

Variable	β [95% CI]	p-value
BMI (kg/m²)	211.0 [90.1, 331.9]	0.0009
Age at OGTT (years)	51.4 [-84.9, 187.7]	0.4547
TSH (mU/L)	106.7 [-417.6, 630.9]	0.6861
Total testosterone (nmol/L)	1664.4 [-739.7, 4068.5]	0.1717
Free testosterone (pmol/L)	83.5 [-1.6, 168.6]	0.0543
Androstenedione (ng/mL)	596.0 [-71.4, 1263.5]	0.0792
AMH (µg/L)	-16.0 [-331.6, 299.7]	0.9197
Cycle day at OGTT	30.9 [10.1, 51.7]	0.0042
Severity of menstrual disorder	906.8 [-1559.2, 3372.7]	0.4655
Metformin use	83.8 [-2061.0, 2228.6]	0.9381
Thyroid hormone therapy	694.0 [-1555.5, 2943.4]	0.5403
Beta-blocker use	2784.5 [-801.5, 6370.5]	0.1259
Progesterone	-131.7 [-350.9, 87.6]	0.2350
17-OH-Progesterone	-0.1 [-10.4, 10.3]	0.9914
Estradiol	-9.8 [-21.5, 1.9]	0.0984
LH	-142.8 [-355.2, 69.7]	0.1845
FSH	-173.0 [-376.9, 30.9]	0.0950
LH/FSH ratio	-646.4 [-1886.1, 593.4]	0.3019
SHBG	-1.0 [-13.2, 11.2]	0.8692
DHEA-S	6.6 [-1.3, 14.6]	0.1011
HbA1c (%)	4138.6 [1172.4, 7104.9]	0.0069
Ferriman-Gallwey score	-49.4 [-1010.7, 911.8]	0.9186

β = regression coefficient. CI = confidence interval. p values are two-sided. HOMA-IR = homeostasis model assessment of insulin resistance; AUC = area under the curve.

Table S9: Change-in-estimate analysis

9.1 Change-in-estimate analysis for log(Matsuda)

Additional variable	BMI effect estimate	Std. Error	p-value	Percent change vs. main model
Parsimonious main model	-0.078	0.010	<0.0001	0.0%
Cycle day at OGTT	-0.073	0.010	<0.0001	-6.7%
Severity of menstrual disorder	-0.076	0.010	<0.0001	-3.2%
Metformin use	-0.079	0.010	<0.0001	+0.5%
Thyroid hormone therapy	-0.080	0.010	<0.0001	+2.2%
Beta-blocker use	-0.079	0.010	<0.0001	+1.0%
Progesterone	-0.080	0.010	<0.0001	+1.6%
17-OH-Progesterone	-0.080	0.010	<0.0001	+1.5%
Estradiol	-0.078	0.010	<0.0001	-0.6%
LH	-0.078	0.010	<0.0001	-0.8%
FSH	-0.079	0.011	<0.0001	+1.2%
LH/FSH ratio	-0.079	0.010	<0.0001	+1.0%
SHBG	-0.082	0.010	<0.0001	+5.1%
DHEA-S	-0.078	0.010	<0.0001	-0.1%
HbA1c (%)	-0.076	0.010	<0.0001	-3.3%
Ferriman-Gallwey score	-0.080	0.010	<0.0001	+2.4%

9.2. Change-in-estimate analysis for log(HOMA-IR)

Additional variable	BMI effect estimate	Std. Error	p-value	Percent change vs. main model
Parsimonious main model	0.094	0.012	<0.0001	0.0%
Cycle day at OGTT	0.088	0.012	<0.0001	-5.8%
Severity of menstrual disorder	0.089	0.012	<0.0001	-4.3%
Metformin use	0.093	0.012	<0.0001	-1.1%
Thyroid hormone therapy	0.095	0.012	<0.0001	+1.2%
Beta-blocker use	0.094	0.012	<0.0001	+0.7%
Progesterone	0.095	0.012	<0.0001	+1.6%
17-OH-Progesterone	0.094	0.012	<0.0001	+0.7%

Additional variable	BMI effect estimate	Std. Error	p-value	Percent change vs. main model
Estradiol	0.093	0.012	<0.0001	-1.0%
LH	0.093	0.012	<0.0001	-0.9%
FSH	0.095	0.013	<0.0001	+2.0%
LH/FSH ratio	0.094	0.012	<0.0001	+0.2%
SHBG	0.097	0.012	<0.0001	+3.3%
DHEA-S	0.093	0.012	<0.0001	-0.2%
HbA1c (%)	0.091	0.012	<0.0001	-3.2%
Ferriman-Gallwey score	0.094	0.012	<0.0001	+0.9%

9.3 Change-in-estimate analysis for AUCglucose$_{(0–180)}$

Additional variable	BMI effect estimate	Std. Error	p-value	Percent change vs. main model
Parsimonious main model	188.8	64.4	0.0047	0.0%
Cycle day at OGTT	177.4	63.2	0.0065	-6.0%
Severity of menstrual disorder	192.8	64.6	0.0040	+2.1%
Metformin use	206.6	63.9	0.0019	+9.4%
Thyroid hormone therapy	205.9	64.8	0.0023	+9.0%
Beta-blocker use	198.5	63.3	0.0026	+5.1%
Progesterone	198.0	63.6	0.0027	+4.9%
17-OH-Progesterone	203.0	63.6	0.0022	+7.5%
Estradiol	184.6	64.1	0.0054	-2.2%
LH	186.6	64.1	0.0049	-1.2%
FSH	179.7	65.5	0.0078	-4.9%
LH/FSH ratio	197.2	63.3	0.0027	+4.4%
SHBG	212.6	64.7	0.0016	+12.6%
DHEA-S	189.6	63.5	0.0040	+0.4%
HbA1c (%)	169.1	62.6	0.0088	-10.5%
Ferriman-Gallwey score	212.6	64.2	0.0015	+12.6%

References

1. Matsuda, M. and R.A. DeFronzo, *Insulin sensitivity indices obtained from oral glucose tolerance testing: comparison with the euglycemic insulin clamp.* Diabetes Care, 1999. **22**(9): p. 1462-70.
2. Lorenzo, C., et al., *Fasting and OGTT-derived measures of insulin resistance as compared with the euglycemic-hyperinsulinemic clamp in nondiabetic Finnish offspring of type 2 diabetic individuals.* J Clin Endocrinol Metab, 2015. **100**(2): p. 544-50.
3. Matthews, D.R., et al., *Homeostasis model assessment: insulin resistance and beta-cell function from fasting plasma glucose and insulin concentrations in man.* Diabetologia, 1985. **28**(7): p. 412-9.
